# Targeting TUBB2B inhibits triple-negative breast cancer growth and brain-metastatic colonization

**DOI:** 10.1186/s13046-025-03312-y

**Published:** 2025-02-17

**Authors:** Qingling He, Jianyang Hu, Fung-Yin Ngo, Huiqi Zhang, Lin He, Hao Huang, Tan Wu, Yilin Pan, Zihan Yang, Yuanyuan Jiang, William C. Cho, Wah Cheuk, Gary M. Tse, Julia Y. Tsang, Mengsu Yang, Liang Zhang, Xin Wang, Pui-Chi Lo, C. Geoffrey Lau, Y. Rebecca Chin

**Affiliations:** 1 Department of Biomedical Sciences and Tung Biomedical Sciences Centre, City University of Hong Kong, Kowloon Tong, Hong Kong; 2 Department of Precision Diagnostic and Therapeutic Technology, City University of Hong Kong Shenzhen Futian Research Institute, Shenzhen, Guangdong China; 3 Department of Neuroscience, City University of Hong Kong, Kowloon Tong, Hong Kong; 4 Key Laboratory of Biochip Technology, Biotech and Health Centre, Shenzhen Research Institute of City University of Hong Kong, Shenzhen, 518057 China; 5 Department of Clinical Oncology, Queen Elizabeth Hospital, Kowloon Tong, Hong Kong; 6 Department of Pathology, Queen Elizabeth Hospital, Kowloon Tong, Hong Kong; 7 Department of Anatomical and Cellular Pathology, State Key Laboratory of Translational Oncology, Prince of Wales Hospital, The Chinese University of Hong Kong, Sha Tin, Hong Kong; 8 Department of Surgery, The Chinese University of Hong Kong, Sha Tin, Hong Kong; 9 Li Ka Shing Institute of Health Sciences, The Chinese University of Hong Kong, Sha Tin, Hong Kong

**Keywords:** Triple-negative breast cancer, TUBB2B, Brain metastatic colonization, Protein translation

## Abstract

**Background:**

The triple-negative subtype of breast cancer is particularly challenging to treat due to its aggressiveness with a high risk of brain metastasis, and the lack of effective targeted therapies. Tubulin beta 2B class IIb (TUBB2B), a β-tubulin isoform regulating axon guidance during embryonic development, was found to be overexpressed in various types of cancers including triple-negative breast cancer (TNBC). However, its functional roles in breast cancer or metastasis remain unclear.

**Methods:**

To identify TUBB2B as a novel molecular target in TNBC, we performed bioinformatics analysis to assess the association of TUBB2B expression and survival of patients. RNAscope in situ hybridization was used to examine TUBB2B expression in clinical breast tumor samples. The effect of TUBB2B knockdown on TNBC growth and brain metastasis colonization was evaluated by in vitro and in vivo assays. Mass spectrometry (MS) and biochemical experiments were performed to explore the underlying mechanisms. Preclinical efficacy of targeting TUBB2B was determined in xenograft studies using the siRNA-gold nanoparticle (siRNA-AuNP) approach.

**Results:**

TUBB2B, but not other β-tubulin isoforms, is frequently overexpressed in TNBC primary tumors as well as brain metastases. We also find that upregulation of TUBB2B is associated with poor prognosis in breast cancer patients. Silencing TUBB2B induces tumor cell death and inhibits the outgrowth of brain metastasis. Mechanistically, we identify eukaryotic translation elongation factor 1 alpha 1 (eEF1A1) as a novel interacting partner of TUBB2B, revealing a previously unexplored role of TUBB2B in translational regulation. In line with its neural-related functions, TUBB2B overexpression in TNBC cells activates astrocytes, which in turn upregulate TUBB2B in tumor cells. These findings suggest a feed-forward interaction between TUBB2B in TNBC cells and astrocytes that promotes brain metastatic colonization. Furthermore, we demonstrate the potent inhibition of TNBC xenograft growth as well as brain metastatic colonization using TUBB2B siRNA-AuNP treatment, indicating potential clinical applications of targeting TUBB2B for TNBC.

**Conclusions:**

TUBB2B is a novel TNBC gene that plays a key role in promoting tumor cell survival and brain metastatic colonization, and can be targeted by siRNA-AuNPs as a treatment strategy.

**Supplementary Information:**

The online version contains supplementary material available at 10.1186/s13046-025-03312-y.

## Introduction

Breast cancer remains the second most common cause of death among women worldwide, accounting for more than 670,000 deaths in 2022 (World Health Organization). Triple-negative breast cancer (TNBC), which lacks the expression of estrogen receptor and progesterone receptor as well as human epidermal growth factor receptor 2 (HER2) amplification, contributes to 15–20% of breast cancer [1]. TNBC is highly aggressive and often spreads to the brain at an earlier stage compared to other breast cancer subtypes [2]. Chemotherapy, usually with high toxicity, is the main treatment option for TNBC. However, despite the ability of some chemotherapeutic agents to cross the blood-brain barrier (BBB), brain metastases show pronounced resistance [3]. Accordingly, there is an unmet need to identify novel molecular targets to circumvent TNBC growth and brain metastatic colonization.

α/β-tubulins are microtubule proteins that play critical roles in intracellular transport and mitosis, and are implicated in cancer progression. The clinical success of many chemotherapeutic drugs has primarily been attributed to their ability to induce mitotic catastrophe via binding to β-tubulin in tumor cells [4]. It is known that there are 9 β-tubulin isoforms. Despite their high amino acid sequence homology, non-redundant roles of the isoforms were identified in different types of cancer. Tubulin beta 3 class III (TUBB3), but not tubulin beta class I (TUBB) or tubulin beta 2 A-C class IIa, IIb, IIc (TUBB2A-C), plays critical roles in pancreatic tumor growth and metastasis [5]. Recently, tubulin beta 4B class IVb (TUBB4B, also known as TUBB2C) was shown to promote non-alcoholic fatty liver disease-associated hepatocellular carcinoma (NAFLD-HCC) [6]. Furthermore, a novel inhibitor targeting βIII- and βIV-tubulins was demonstrated to effectively inhibit pancreatic cancer growth [4], highlighting the potential of precisely targeting a specific β-tubulin isoform for cancer treatment.

Tubulin beta 2B class IIb (TUBB2B), a β-tubulin isoform primarily expressed in the brain during the embryonic stage, plays a key role in axon guidance during development [7]. Loss-of-function mutations in TUBB2B result in congenital neuronal disorders [8]. In the context of cancer, recent studies reported overexpression of TUBB2B in hepatocellular carcinoma (HCC), neuroblastoma, Hodgkin lymphoma and endocrine therapy-resistant breast cancer [9, 10]. TUBB2B has also been found to be overexpressed in the brain metastases of TNBC patients [11]. Moreover, TUBB2B was one of 48 proteins exclusively found in exosomes of TNBC but not normal breast cells [12]. Higher TUBB2B expression in a subpopulation of endometrial cancer patients is associated with poorer prognosis [13]. Additionally, TUBB2B has been shown to promote HCC growth by regulating cholesterol metabolism [14]. However, the functional role of TUBB2B in the pathogenesis of breast cancer or metastasis has yet to be studied.

Here, we report that TUBB2B expression is enriched in TNBC primary tumors and brain metastases. We also observed an association between increased TUBB2B expression and unfavorable overall survival (OS) as well as distant metastasis-free survival (DMFS) outcomes in breast cancer patients. Silencing TUBB2B inhibits TNBC spheroid growth and promotes apoptosis. Depletion of TUBB2B reduces the growth of TNBC primary tumors and brain colonization in vivo. Mechanistically, we demonstrate a novel function of TUBB2B where it interacts with eukaryotic translation elongation factor 1 alpha 1 (eEF1A1) and promotes protein synthesis by stabilizing this translation elongation factor. In the brain tumor microenvironment, TUBB2B activates astrocytes, which in turn upregulate expression of TUBB2B in TNBC cells. Finally, we developed TUBB2B siRNA-gold nanoparticles (siRNA-AuNPs) and showed a significant inhibition of TNBC growth and brain metastatic colonization in xenograft models. Thus, we identified TUBB2B as a clinically relevant molecular target for treating TNBC.

## Materials and methods

### Cell culture

HCC1806, BT549, HCC1143, and HEK293T cells were obtained from American Type Culture Collection (ATCC). Human mammary epithelial cells (HMEC) were purchased from Lonza Bioscience. Astrocytes were purchased from Cell Applications (# 882 A-05a). HCC1806, BT549, and HCC1143 cells were maintained in Roswell Park Memorial Institute 1640 medium (RPMI; Gibco) containing 10% fetal bovine serum (FBS; Clontech). HEK293T cells were cultured in Dulbecco’s Modified Eagle medium (DMEM; Gibco) containing 10% FBS. HMEC was cultured in the Mammary Epithelial Cell Growth Medium (cat # CC-3150) specified by the supplier (Lonza Bioscience). Astrocytes were maintained using the Human Astrocyte Growth Medium Kit (Cell Applications, 821 K-500) and subcultured using Subculture Reagent Kit (Cell Applications, 090 K). All cell lines are regularly tested negative for mycoplasma. They have been tested for authentication using short tandem repeat profiling and passaged for < 6 months.

### Lentiviral vector infection

The short hairpin RNAs (shRNAs) (Supplementary Table 1) specifically targeting TUBB2B mRNA were ligated to the lentiviral vector pLKO.1 or pLKO.1-tet-on digested by AgeI (NEB, R3552S) and EcoRI (NEB, R3101s) restriction enzymes to generate plasmids (pLKO.1-shRNA1/shRNA2-TUBB2B, pLKO.1-tet-on-shRNA1/shRNA2-TUBB2B). For overexpression of exogenous eEF1A1, the coding DNA sequence (CDS) of eEF1A1 was synthesized and cloned into vector CD532A-1 by GENEWIZ. To prepare lentiviral supernatants, 10 µg lentiviral vectors (pLKO.1-shRNA CTL, pLKO.1-sh1-TUBB2B, pLKO.1-sh2-TUBB2B, pLKO.1-tet-on-shRNA CTL, pLKO.1-tet-on-sh1-TUBB2B, pLKO.1-tet-on-sh2-TUBB2B, CD532A-1, CD532A-1-eEF1A1) were co-transfected with 7 µg psPAX2 and 2.4 µg VSV-G vectors into HEK293T cells in a 10 cm plate, using polyethylenimine as the transfection reagent. Sixty hours post transfection, the supernatants containing lentiviruses were collected and filtered using 0.45 μm syringe filters (Thermo Fisher 7232545). For infection of lentivirus to TNBC cells, 0.15–0.2 ml lentivirus with 5 µg/ml polybrene was added to cells precultured in a 6-well plate. After 48–60 h of infection with lentivirus, cells were used for protein and RNA extraction or downstream functional assays.

### Reverse transcription-quantitative PCR (RT-qPCR)

Total RNA was extracted using the RNeasy Plus Mini Kit (Qiagen #74134) according to the manual of the kit. Taqman Reverse Transcription Reagent (Applied Biosystems, N8080234) was used for the reverse transcription. RT-qPCR was processed using a QuantStudio 12 K Flex Real-time PCR System (Applied Biosystems). All primers used in the RT-qPCR are listed in Supplementary Table 1.

### CellTiter-Glo® three-dimensional (3D) and two-dimensional (2D) cell viability assays

Cell viability in 3D and 2D cultures was determined as previously described [15]. Briefly, for 3D cultures, growth factor-reduced Matrigel (Corning) was used to coat 96-well plates (Corning # 3610). Cells were seeded onto the precoated 96-well plates in assay medium (relevant complete medium with 2% Matrigel supplement), with a density of 2000 (for BT549 and HMEC) or 1000 (for HCC1806) cells per well and allowed to grow in a 5% CO_2_ humidified incubator at 37 °C for 7 days to form spheroids. For inducing knockdown in spheroids, 3 days after seeding the cells in 3D culture, doxycycline (dox) was added at a final concentration of 100 ng/µl and the spheroids were allowed to grow for another 7 days. Assay medium with or without dox was added every 3–4 days. To quantify 3D spheroid viability, the CellTiter-Glo^®^ 3D Cell Viability Assay (Promega #G9682) was performed following the instructions of the kit manual. For the assessment of 2D cell viability, cells were seeded onto a 96-well plate, with a density of 4000 (for BT549 and HMEC) or 2000 (for HCC1806) cells per well, and cultured for 3 days. The CellTiter-Glo^®^ Luminescent Cell Viability Assay (Promega #G7571) was used to quantify 2D cell viability according to the manufacturer’s instructions. The bioluminescence signal was read by the BioTek Synergy™ H1 Microplate Reader.

### Western blot (WB)

Total protein was extracted by lysing cells in EBC buffer (0.5% NP-40, 120 mM NaCl, 50 mM Tris-HCl (pH 7.4),50 nM calyculin, 1 mM sodium pyrophosphate, 20 mM sodium fluoride, 2 mM EDTA, 2 mM EGTA, proteinase inhibitor cocktail) on ice for 30 min. The protein concentration in the pre-cleared cell lysates was measured by the Bio-Rad protein assay reagent and read by the BioTek Synergy™ H1 Microplate Reader. Protein samples (10–20 µg) were separated by sodium dodecyl sulfate-polyacrylamide gel electrophoresis (SDS-PAGE) and transferred to a nitrocellulose membrane at 280 mA for 90 min. After blocking in 5% fat-free milk in TBST buffer (10 mM Tris-HCl (pH 8.0), 150 mM NaCl, 0.2% Tween-20) at room temperature for 1 h, blots were probed with the relevant primary antibodies at 4 °C overnight with gentle rocking. The blots were washed in TBST and then incubated with horseradish peroxidase (HRP)-conjugated secondary antibodies for 1 h at room temperature. After washing 3 times in TBST, membranes were visualized using an enhanced chemiluminescence substrate (Pierce). The primary antibodies used were anti-TUBB2B (sigma, T8453), anti-eEF1A1 (Proteintech, 11402-1-AP), anti-poly(ADP-ribose) polymerase 1 (PARP) (CST, 9542T), anti-caspase 3 (CST, 14220), anti-cleaved-caspase 3 (CST, 9664 S), anti-caspase 8 (CST, 4790), anti-cleaved-caspase 8 (CST, 9748), anti-p-mixed lineage kinase domain like pseudokinase (pMLKL) (CST, 91689 S), anti-MLKL (Santa Cruz, sc-293201), and anti-β-actin (CST, 3700 S).

### Clonogenic growth assays

Cells were seeded in a 6-well plate at a density of 2000 cells per well and cultured for 2 weeks. The medium was changed every 4 days. After 2 weeks, cells were fixed with 4% formaldehyde for 15 min at room temperature. Colonies were stained with 0.1% crystal violet for 40 min followed by a phosphate-buffered saline (PBS) wash. Pictures were captured, and the colony number was counted using the ImageJ software.

### Annexin V and propidium iodide (PI) analysis

1 × 10^6^ cells in PBS were incubated with Annexin V (Invitrogen, 88-8103-72) or PI (Invitrogen, P3566) respectively on ice for 30 min in the dark. After washing, the stained cells were analyzed by Beckman Coulter CytoFLEX S Flow cytometer analyzer.

### RNA in situ hybridization (RISH) of clinical breast cancer samples

Breast or brain metastasis tissue samples were obtained from breast cancer patients who underwent biopsy or surgery for resection at Queen Elizabeth Hospital. Formalin-fixed paraffin-embedded (FFPE) sections were prepared at 5 μm. RISH on these FFPE slides was performed using the RNA scope kit (cat #322370, Advanced Cell Diagnostics) according to the manufacturer’s instructions. Briefly, slides were deparaffinized and then treated with hydrogen peroxide for 10 min. Antigen retrieval was performed by boiling the sections in pre-heated 98–102 °C target retrieval reagent. After being treated with protease for 30 min, probe hybridization and signal amplification steps were performed according to the manual instructions. Slides were counterstained with hematoxylin and coverslipped. Probes for TUBB2B (cat # 1215181-C1), a positive control probe for a housekeeping gene (PPIB, cat # 313901), and a negative control probe for DapB, an E.coli gene (cat # 310043), were purchased from Bio-Techne. The stained sections were semi-quantitatively scored according to their staining intensity with negative staining scored as grade 0/1 and positive staining scored as grade 2–4. The procedures were approved by the Human Subjects Ethics Committees at City University of Hong Kong and conformed to government regulations for research involving human participants. Informed consent was obtained from the breast cancer patients.

### Immunofluorescence (IF)

Tumor spheroids grown in Matrigel in chamber slides or 2D cells grown on glass-bottom slides were fixed with 4% paraformaldehyde for 20 min, followed by 0.5% Triton X-100 for 2 min. Then cells were rinsed with 100 mmol/L glycine in PBS three times. Cells were blocked with 10% goat serum in IF buffer (130 mmol/L NaCl, 7 mmol/L Na_2_HPO_4_, 3.5 mmol/L NaH_2_PO_4_, 7.7 mmol/L NaN_3_, 0.1% BSA, 0.2% Triton X-100, and 0.05% Tween-20) for 1 h at room temperature, and probed with primary antibodies (anti-active-caspase 3, anti-glial fibrillary acidic protein (GFAP)) overnight at 4 °C. After washing with IF buffer three times, cells were incubated with Alexa Fluor 647-conjugated Goat Anti-Rabbit IgG (Jackson Immuno Research, 111-605-003) or Alexa Fluor 488-conjugated Goat Anti-Rabbit IgG for 45 min. Nuclei were stained with Hoechst. For visualizing F-actin, Alexa Fluor 488–conjugated phalloidin (Invitrogen) was applied together with Hoechst. Cells were then rinsed with IF buffer and PBS and mounted with prolong anti-fade. Images were captured using confocal fluorescence microscopy. The fluorescence density was quantified with ImageJ software.

### Co-immunoprecipitation (co-IP) and mass spectrometry (MS) analysis

Cells grown on 10 cm cell culture plates were washed twice with PBS and then applied with 1 ml of EBC lysis buffer with protease inhibitors. Cell lysate was transferred to Eppendorf tubes and placed on ice for 30 min. Following a centrifugation at 13,000 g for 20 min at 4 °C, the supernatant fraction (1 mg protein) was incubated with indicated antibodies at 4 °C overnight. Then, 15 µl of Dynabeads™ Protein G beads (Invitrogen, 10003D) were added, followed by incubation at 4 °C for 2 h. After washing the beads 4 times with lysis buffer, bound proteins were eluted with 50 µl SDS gel loading buffer. The eluates were separated by SDS-PAGE. For the samples for WB analysis, the protein was transferred to a nitrocellulose membrane, followed by detection with the indicated antibodies. For the samples for mass spectrum analysis, the gel was stained with SYPRO Ruby Protein Gel Stain solution (Invitrogen) and scanned by ChemiDoc using the Ruby stain channel. Protein bands with differentiated signal were excised from the gel and digested with sequencing-grade trypsin (Promega) dissolved in 50 mM ammonium bicarbonate buffer overnight at 37 °C. The digested samples were analyzed on a platform with an Easy-nLC 1200 chromatography system (Thermo Scientific) coupled to a Q Exactive HF mass spectrometry machine (Thermo Scientific). The XCalibur 4.0.27 (Thermo Scientific) software was used to obtain the raw data, which were processed with Proteome Discoverer software suite version 2.2 (Thermo Scientific), against a UniProt human refseq database using Sequest HT.

### Puromycin incorporation assay

HCC1806 or BT549 cells were seeded at 2–5 × 10^5^ cells in 6-well plates 24 h prior to relevant lentivirus infection. Forty-eight hours after lentivirus infection, puromycin pulses were performed by incubating the cells with 10 µg/mL puromycin for 15 min at 37 °C. Then, the cells were washed with pre-cold PBS and lysed in EBC buffer. 10 µg of protein was assayed for WB analysis using the anti-puromycin antibody (Millipore, MABE343).

### Xenograft studies

Female nude or NOD scid gamma (NSG) mice (6–8 weeks old) were purchased from the Laboratory Animal Research Unit, City University of Hong Kong. All procedures followed the protocol approved by the Animal Ethics Committees at City University of Hong Kong and conformed to government guidelines for the care and maintenance of laboratory animals. The maximal tumor size permitted by the Animal Ethics Committees is 2 cm in diameter. It is confirmed that in this study, the maximal tumor size in mice was not exceeded. Randomization was used to allocate experimental units to control and experimental groups.

For breast cancer xenografts in mammary fat pad (MFP), 2 × 10^6^ HCC1806 cells expressing control or TUBB2B shRNA and/or eEF1A1 overexpression were resuspended in 50% matrigel in PBS and implanted into bilateral orthotopic MFP of nude mice. Tumor formation was examined every 3–5 days. For dox-induced shRNAs expression, 2 × 10^6^ HCC1806 cells expressing tet-on-shRNA-TUBB2B were implanted into MFP. Mice were administered dox at day 11 after injection. All mice were euthanized, and tumors were collected at the end of experiments.

For brain metastasis, 6 × 10^4^ HCC1806 cells expressing Firefly luciferase were diluted in 10 µl PBS and injected into the internal carotid arteries (ICA). The brain metastasis progression was monitored using an IVIS animal imaging (PerkinElmer) once a week. The luminescence was quantified at the indicated time points.

### Cytokine and chemokine profiling

Human astrocytes were incubated in complete RPMI medium with or without HCC1806-conditioned medium (HCC1806-CM). At 72 h after culture, the conditioned medium was harvested and processed for cytokine and chemokine profiling using the human cytokine array kit (R&D system, ARY005B), which detects 36 cytokines, chemokines, and soluble mediators. The array membranes were probed with the mixture of conditioned medium and the antibody cocktail overnight at 4 °C. After several washing, the membranes were incubated with secondary antibodies conjugated with HRP. The membranes were then exposed to the HRP substrate. The intensity of the reaction was quantified on ImageJ software.

### Gold nanoparticle (AuNP) synthesis and evaluation

AuNPs loaded with siRNA were synthesized according to the published procedures [16]. In brief, thiol-modified RNA duplexes were added to RNase-free AuNPs and incubated for 45 min at room temperature. Subsequently, the concentration of NaCl was increased to 0.3 M to allow conjugation and the mixture was incubated for 16 h at room temperature. The siRNA-functionalized AuNPs were then treated with 30 µM of polyethylene glycol-Thiol (PEG-SH) for 6 h as an additional surface backfill. The unbound agents were removed by centrifugation and the pellets were washed in RNase-free water twice. The final pellets were resuspended in PBS and stored at 4 °C. For in vitro administration, the siRNA-AuNPs were added directly to the medium of TNBC cells at a concentration of 100 nM for 48 h. To visualize siRNA-AuNP uptake, we plated the cells on a glass-bottom plate and counterstained with Hoechst after treatment with siRNA-AuNP and subjected them to confocal fluorescence microscopy. To assess functionality, the treated cells were collected for RT-qPCR with TUBB2B primers, 2D cell titer assay, and 3D cell titer assay with or without astrocyte coculture. For in vivo administration, orthotopic tumors were formed by injecting TNBC cells into MFP or via ICA to the brain for 11 days. Then, siRNA-AuNPs were administered via tail vein at a volume that allowed for 1.4 mg/kg (RNA/mouse weight) per injection, with injections performed daily or every other day.

### Bioinformatics analyses

To access the expression levels of TUBB2B and other β-tubulin isoforms in breast cancer subtypes, the Cancer Genome Altlas (TCGA) dataset was accessed through the cBioPortal database (https://www.cbioportal.org/). mRNA levels of β-tubulin isoforms in tumors were compared to matched normal tissue from the same patient. OncoPrint graphs of different subtypes were shown with mRNA high (red), mRNA low (blue), and no alteration (grey). The mRNA high percentage for each subtype was then calculated.

To evaluate the expression levels of TUBB2B and other β-tubulin isoforms in different metastatic sites, the clinical information and normalized mRNA expression data were downloaded from the Gene Expression Omnibus (GEO) (https://www.ncbi.nlm.nih.gov/gds). The expression levels were visualized by GraphPad Prism (version 9.2).

To evaluate the OS and DMFS of breast cancer patients, we used the Kaplan-Meier survival analysis. Clinical information and transcriptomic data were obtained from TCGA, and from GEO. The survival curves were generated using the GraphPad Prism (version 9.2). Patients were stratified into high and low expression groups based on the auto selected best cutoff of the gene of interest. The log-rank test was used to determine the statistical significance of differences between survival curves.

### Microfluidic device for coculture studies

The fabrication protocol for the microfluidic device has been previously described [17]. The device was designed using AutoACD software, and a mask film was printed. The master mold was created using SU-8 3005 and 3050 (Kayakuam, Japan) at spin speeds of 2500 and 3000 rpm, respectively. This process involved a series of soft-lithography steps, including pre-baking, exposure, post-baking, and development, to produce micrometer-scale patterns on silicon wafers. The devices were then molded using poly(dimethylsiloxane) (PDMS) (a 10:1 mixture of silicone elastomer and curing agent, Sylgard 184, Dow Corning, Midland, MI) against the master mold and degassed in a vacuum tank for 15 min. Subsequently, the molds were incubated at 65 °C for 2 h to ensure complete curing of the PDMS. Once cured, the PDMS patterns were peeled off, and channel inlets and outlets were punched using a 2 mm diameter circular hole puncher. The PDMS layers were treated with air plasma (Plasma cleaner/sterilizer, PPC-3XG, Harrick, NY, US). Following fabrication, the device was sterilized by exposure to UV light for 45 min. The sterilized PDMS device was then bonded to a cover glass. To enhance cell adhesion, fibronectin (2%) (ThermoFisher, USA) was added to the device channel and incubated at 37 °C. A mixture of cancer cells and Matrigel was introduced into the device inlet, allowing the cells to load into the chamber. After three days, during which the cancer cells formed spheroids, astrocytes were added to the channels on either side. Images were captured using a light microscope.

### Statistical analysis

Statistical significance between conditions was assessed by Student’s t-tests. In all the figures, data are presented as mean ± standard error of the mean (SEM). Significance between conditions is denoted as *p < 0.05, **p < 0.01, and ***p < 0.001. At least three independent experiments were performed for each condition to verify the emphasized trends in in vitro studies.

## Results

### TUBB2B is highly expressed in TNBC primary tumors and brain metastases, and is correlated with poor prognosis

**Fig. 1:**
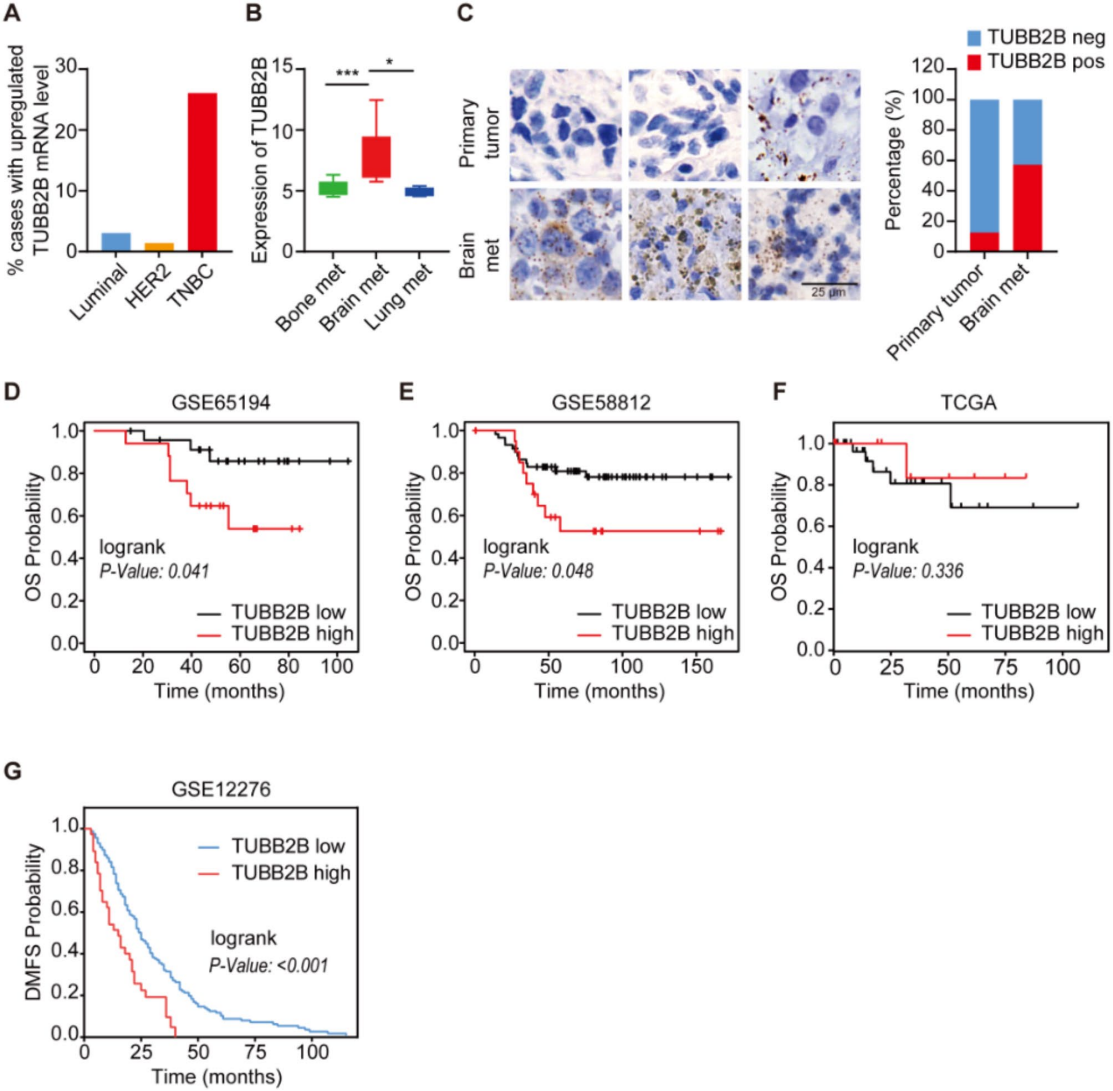
TUBB2B is highly expressed in TNBC and is correlated with poor prognosis. (**A**) Bar graph depicting the percentage (%) of breast tumors with TUBB2B mRNA upregulation, using a dataset from The Cancer Genome Atlas (Luminal: *n* = 228; HER2+: *n* = 120; TNBC: *n* = 82). (**B**) Graph showing higher expression of TUBB2B in brain metastases (met), compared to bone or lung metastases of breast cancer. Data were from the Human Cancer Metastasis Database; GSE14017 (*n* = 29; Lung met: *n* = 4; Brain met: *n* = 15; Bone met: *n* = 10). (**C**) Representative images of RNAscope staining of TUBB2B in TNBC primary (*n* = 8) and TNBC brain metastases (*n* = 7) tissues. The bar graph shows the % of TUBB2B positive (pos) and negative (neg) cases. (**D** and **E**) OS curves for PAM50-basal breast cancer patients with high and low TUBB2B expression. GSE65194 (*n* = 41; TUBB2B low: *n* = 24; TUBB2B high: *n* = 17); GSE58812 (*n* = 80; TUBB2B low: *n* = 59; TUBB2B high: *n* = 21). (**F**) OS curves for TNBC patients from TCGA (*n* = 42) with high (*n* = 12) and low (*n* = 30) TUBB2B expression. (**G**) Kaplan-Meier plots of DMFS probability for breast cancer patients (GSE12276, *n* = 202) with high (*n* = 40) and low (*n* = 162) TUBB2B expression. All error bars represent SEM, two-tailed Student t-test (* *P* < 0.05, *** *P* < 0.001)

In this study, we applied bioinformatics analysis to search for TNBC driver genes that play critical roles in primary tumors as well as brain metastatic colonization. Using clinically annotated gene expression datasets (*n* = 310, TCGA; [18]), we found that TUBB2B mRNA is upregulated in 26% of TNBC cases, compared to 3% and 1.3% in luminal and HER2 + breast tumors, respectively (Fig. 1A). Among 9 β-tubulin isoforms, TUBB2B is also found to be overexpressed in the highest percentage of TNBC cases (TCGA, *n* = 82; Fig. S1A). In breast cancer patients, TUBB2B, but not other β-tubulin isoforms, is upregulated in brain metastases compared to lung and bone metastases (Fig. 1B, Fig. S1B-H; GSE14017). Previous RNA-seq data from clinical samples and xenografts also showed a higher expression of TUBB2B in TNBC brain metastases than in primary tumors [11]. Using RNAscope in situ hybridization, we not only validated the high expression of TUBB2B in 13% of TNBC primary tumor cases, but also observed high levels of TUBB2B in 57% of TNBC brain metastasis cases (Fig. 1C). TUBB2B overexpression is a predictor of poor OS in PAM50-basal breast cancer patients (Fig. 1D (GSE65194), 1E (GSE58812)), although analysis with TNBC patients in the TCGA datasets does not show significance (Fig. 1F). TUBB2B overexpression was also found to be a predictor of poor DMFS in breast cancer patients (Fig. 1G). These data support the clinical relevance of TUBB2B and suggest its oncogenic function in TNBC.

### TUBB2B is required for TNBC spheroid growth and maintenance

**Fig. 2:**
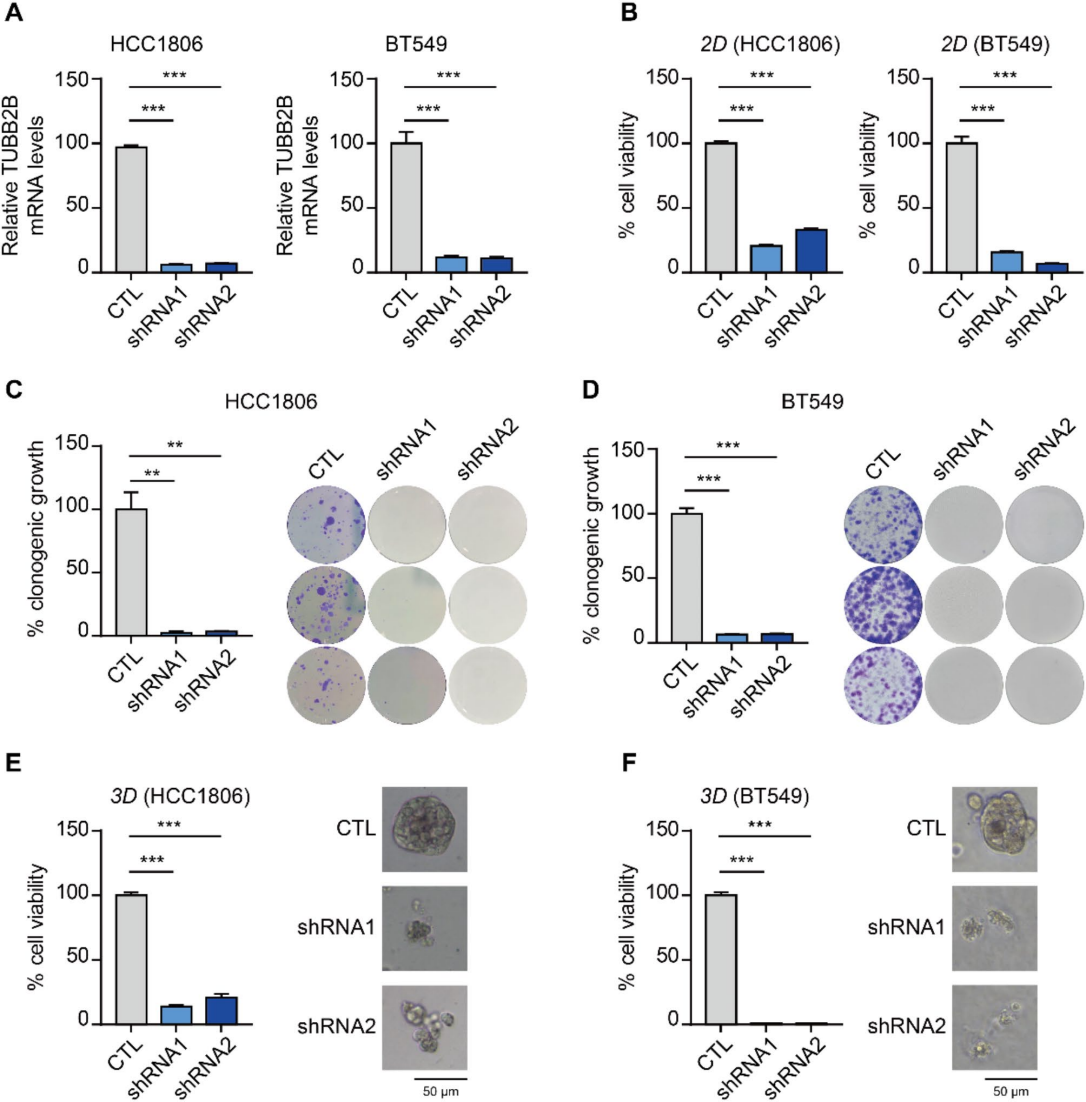
TUBB2B depletion potently reduces TNBC cell viability. (**A**) The mRNA levels of TUBB2B were analyzed by RT-qPCR in HCC1806 and BT549 cells infected with control or TUBB2B shRNAs lentiviral vector. The bar graph depicts the relative TUBB2B mRNA levels to glyceraldehyde-3-phosphate dehydrogenase (GAPDH). (**B**) 2D cell titer-Glo assay of HCC1806 and BT549 cells. (**C** and **D**) HCC1806 and BT549 cells infected with control or TUBB2B shRNAs lentiviral vector were cultured (2000 cells/ well) for clonogenic assay for 14 days. Representative images are shown. (E and F) 3D cell titer-Glo assay for HCC1806 (**E**) and BT549 (**F**) cells expressing control or TUBB2B shRNAs and representative images of 3D spheroids are shown. All error bars represent SEM, two-tailed Student t-test (* *P* < 0.05, ** *P* < 0.01, *** *P* < 0.001)

To explore the function of TUBB2B in TNBC, we generated a panel of breast cancer lines with TUBB2B knockdown. Depletion of TUBB2B mRNA was > 90% with 2 distinct shRNAs (Fig. 2A). We also confirmed the knockdown of TUBB2B by WB analysis (Fig. S2A). Due to the cross-reactivity of anti-TUBB2B antibody to other β-tubulin isoforms [19], RT-qPCR was used in this study to validate the potency and specificity of TUBB2B knockdown. By performing RT-qPCR, we verified that the TUBB2B shRNAs specifically knocked down TUBB2B but not other β-tubulin isoforms (Fig. S2B). TUBB2B silencing resulted in potent inhibition of TNBC viability in 2D cultures of HCC1806 and BT549 cells (Fig. 2B). Furthermore, TUBB2B knockdown significantly reduced colony formation in clonogenic assays (Fig. 2C and D). In line with previous findings that TUBB2B was minimally expressed in normal breast epithelium [20], we found it to be expressed at a low level in luminal breast cancer MCF7 cells and normal HMEC (Fig. S2C). Nevertheless, shRNA effectively depleted TUBB2B in these cells (Fig. S2D). TUBB2B knockdown only had a mild effect on MCF7 cells and did not affect the viability of HMEC (Fig. S2E). Using the 3D spheroid assay, which more accurately recapitulates tumor phenotypes in vivo, we demonstrated that TUBB2B depletion significantly inhibited TNBC cell viability in 3D cultures (Figs. 2E and F and 80–99% inhibition). These findings highlight the therapeutic potential of targeting TUBB2B for TNBC.

### Silencing TUBB2B induces apoptosis in TNBC cells

**Fig. 3:**
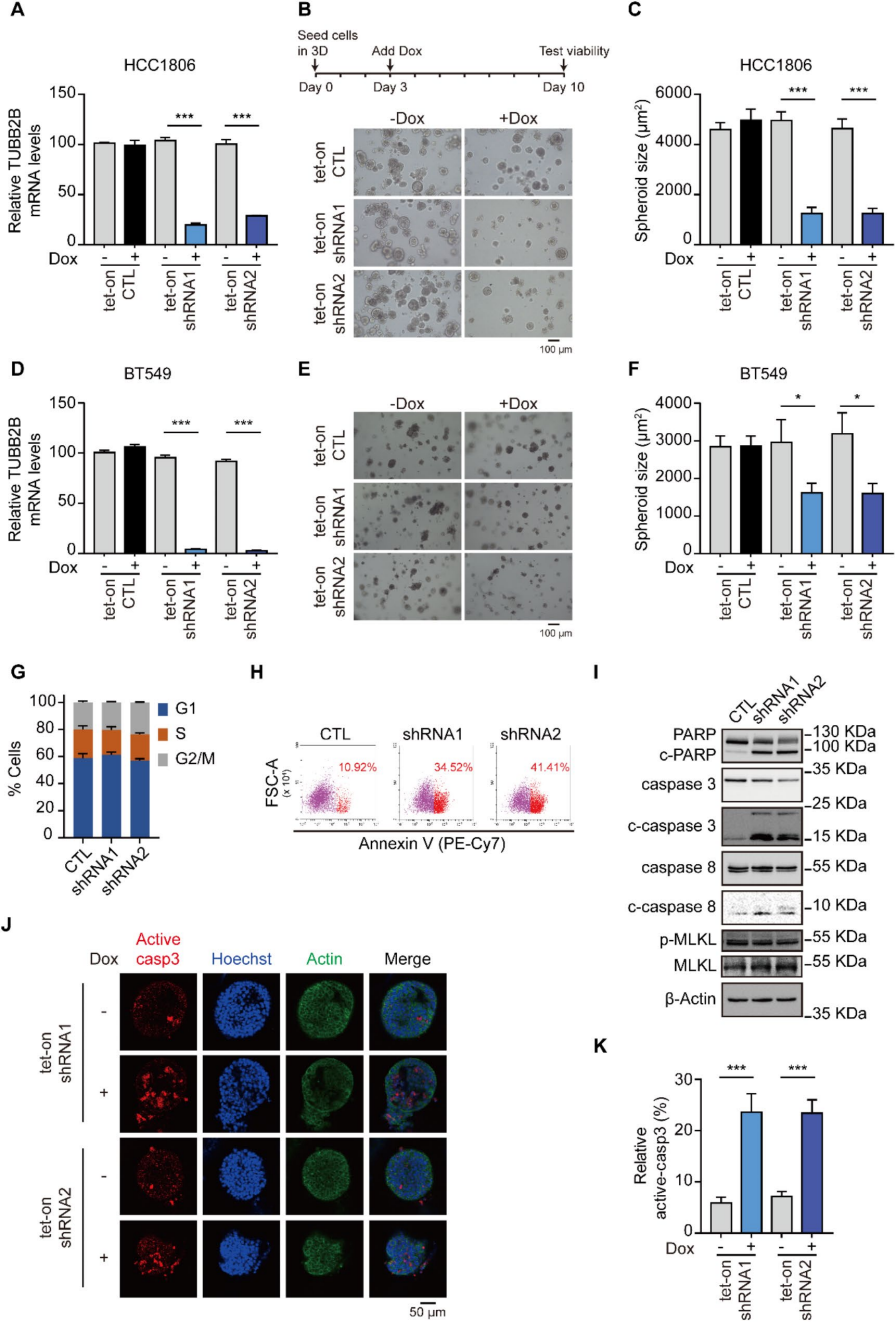
TUBB2B plays a key role in TNBC spheroid maintenance and tumor cell survival. (**A**) HCC1806 cells expressing tet-on control or TUBB2B shRNAs were treated with dox (100 ng/ml) for 3 days. Total mRNA was then collected for RT-qPCR analysis. The bar graph depicts the relative TUBB2B mRNA levels to GAPDH. (**B**) Schematic of 3D culture. Briefly, HCC1806 cells expressing tet-on-control or TUBB2B shRNAs were seeded in 3D for 3 days without dox treatment. Then, at day 3, dox (100 ng/ml) was added to induce TUBB2B depletion. Every 48 h, the medium was changed with or without dox. Representative images at day 10 are shown. (**C**) Bar graphs show the size of HCC1806 spheroids at day 10. (**D**) BT549 cells expressing tet-on control or TUBB2B shRNAs were treated with dox (100 ng/ml) for 3 days. Total mRNA was then collected for RT-qPCR analysis. The bar graph depicts the relative TUBB2B mRNA levels to GAPDH. (**E**) Representative images of BT549 spheroids. (**F**) Bar graphs show the size of BT549 spheroids at day 10. (**G**) BT549 cells containing TUBB2B shRNAs or control were subjected to flow cytometry after PI staining. The ratio of G1, S, G2/M phase is shown. (**H**) BT549 cells containing TUBB2B shRNA or control were stained using Annexin V and subjected to flow cytometry. The ratio of Annexin V-positive cells is shown. (**I**) BT549 cells infected with control or TUBB2B shRNAs were subjected to WB using whole-cell lysates with indicated antibodies. (**J**) HCC1806 cells expressing tet-on TUBB2B shRNAs were grown in 3D for 5 days, followed by dox (100 ng/ml) treatment for 5 days. IF was performed using active caspase-3 (active casp3) antibodies. Nuclei and actin were labelled with Hoechst and fluor 488-conjugated phalloidin, respectively. Images were captured by confocal microscopy. (**K**) The percentage of cells containing active caspase-3 was quantified and depicted in the bar graph. All error bars represent SEM, two-tailed Student t-test (* *P* < 0.05, *** *P* < 0.001)

Next we utilized a tet-on dox-inducible shRNA system to knockdown TUBB2B using two independent shRNAs. This system allows us to deplete genes in a temporal manner, which is particularly useful for studying gene functions in tumor maintenance. In the stable cell lines, TUBB2B could be efficiently knocked down 2 days after dox treatment (Fig. 3A and D). In our tumor spheroid maintenance studies, the spheroids were formed for 3 days in 3D culture, and then treated with dox for another 7 days. Silencing of TUBB2B resulted in the disintegration of spheroids (Fig. 3B and E), and the size of the spheroids significantly diminished upon TUBB2B knockdown (Fig. 3B, C, E and F).

Mechanistically, cell cycle profiling was performed in TUBB2B-depleted cells, considering the known function of tubulin in mitosis. Surprisingly, TUBB2B knockdown had a minimal effect on cell cycle progression (Fig. 3G), suggesting that mitotic catastrophe is unlikely to be the cell death mechanism. Instead, our data showed that TUBB2B silencing induced TNBC cell apoptosis, as assessed by Annexin V staining (Fig. 3H). This was accompanied by Caspase 3/8 activation and increased levels of cleaved PARP (Fig. 3I). Levels of MLKL phosphorylation were not affected by TUBB2B knockdown, indicating that necroptosis is not involved in the cell death mechanism. Using the inducible knockdown strategy, along with staining apoptotic cells with active caspase 3 antibodies and confocal microscopy, we further demonstrated that TUBB2B depletion induced potent apoptosis in TNBC spheroids compared to control spheroids (Fig. 3J and K).

### TUBB2B interacts with eEF1A1 and promotes protein translation

**Fig. 4:**
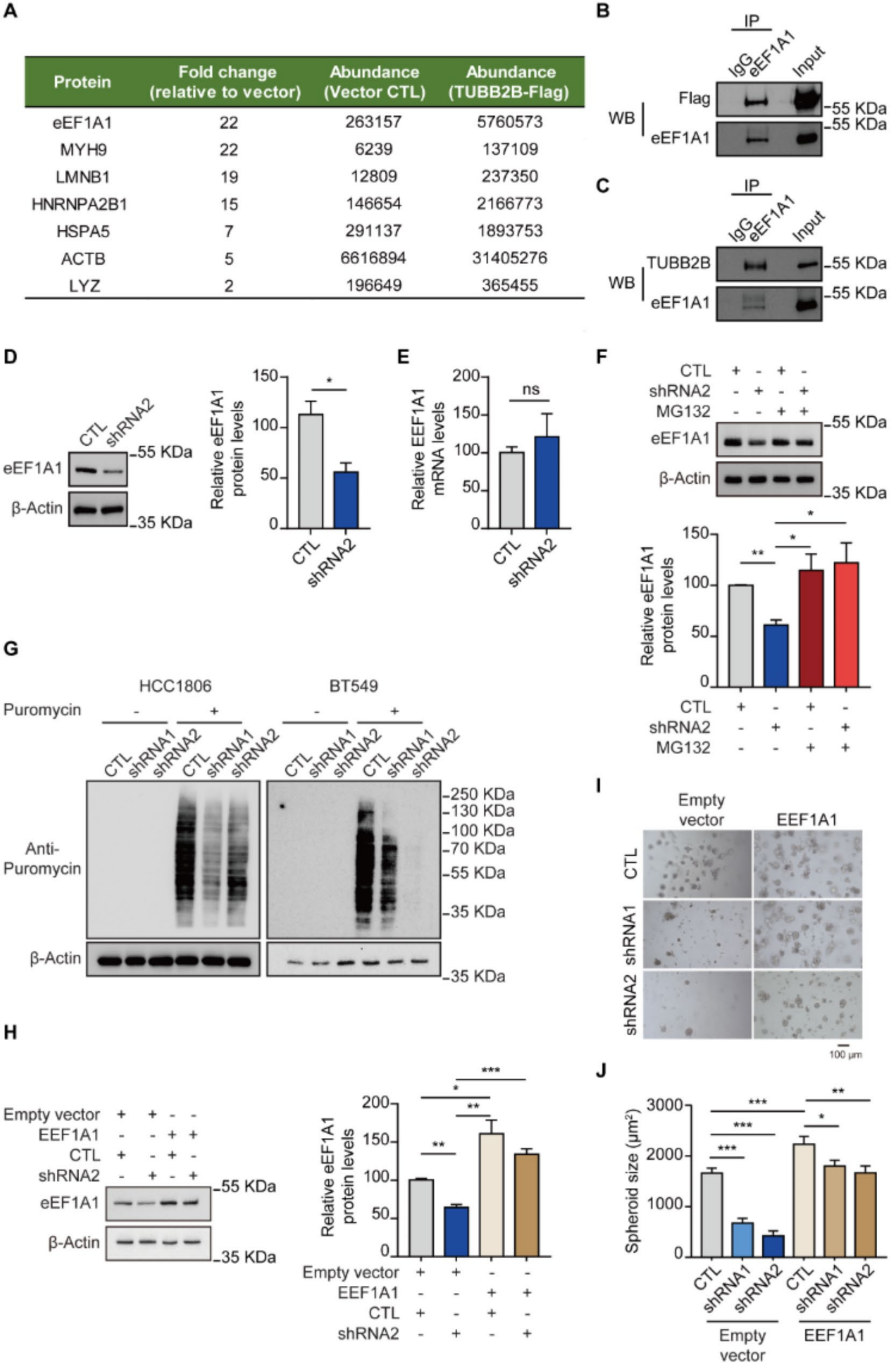
TUBB2B promotes protein translation by interacting with eEF1A1. (**A**) A partial list of TUBB2B-binding proteins identified by MS. (**B**) Co-IP with eEF1A1 antibody in HCC1806 cells overexpressing TUBB2B-Flag, then WB with Flag or eEF1A1 antibody. (**C**) Co-IP with eEF1A1 antibody in HCC1806 wild type (WT) cells, then WB with TUBB2B antibody and eEF1A1 antibody. (**D**) BT549 cells expressing control or TUBB2B shRNA2 were subjected to WB analysis for eEF1A1 protein level. The eEF1A1 protein level was quantified and shown in the bar graph. (**E**) The mRNA level of eEF1A1 was analyzed by RT-qPCR in BT549 cells with control and TUBB2B shRNA2 lentivirus. The bar graph depicts the relative eEF1A1 mRNA level to GAPDH. (**F**) HCC1806 cells containing TUBB2B shRNA2 or control were treated with MG132 (10 µM) for 24 h and then subjected to WB analysis for eEF1A1. The eEF1A1 protein levels were quantified and shown in the bar graph. (**G**) Representative immunoblots of newly synthesized proteins labeled by puromycin in HCC1806 and BT549 cells with control or TUBB2B shRNAs. (**H**) Overexpressing eEF1A1 or CD532A-1 empty vector in HCC1806 cells containing TUBB2B shRNA2 or control and WB analysis for eEF1A1. The eEF1A1 protein levels were quantified and shown in the bar graph. (**I**) Representative images of 3D spheroids of HCC1806 with TUBB2B or control shRNAs in the overexpression of CD532A-1 empty vector or eEF1A1. (**J**) Bar graph shows the spheroid size of HCC806 cells expressing control or TUBB2B shRNAs in the overexpression of CD532A-1 empty vector or eEF1A1. All error bars represent SEM, two-tailed Student t-test (* *P* < 0.05, ** *P* < 0.01, *** *P* < 0.001)

To dissect the molecular mechanisms by which TUBB2B regulates TNBC spheroid growth, we aimed to identify novel TUBB2B-interacting partners by performing immunoprecipitation (IP) followed by MS screening in flag-TUBB2B overexpressing TNBC cells. eEF1A1, an evolutionary conserved translation elongation factor, was one of the most enriched TUBB2B-binding candidates (Fig. 4A). It was prioritized for validation as a TUBB2B-binding partner, because of its critical function in cell proliferation and apoptosis [21] as well as its implications in a variety of cancers, including HCC, B cell lymphoma, colorectal cancer, prostate cancer and breast cancer [22–26]. Our co-IP experiments validated the interactions between eEF1A1 and flag-TUBB2B as well as endogenous TUBB2B in TNBC cells (Fig. 4B and C). Our data further indicate that TUBB2B regulates eEF1A1 protein (Fig. 4D) but not mRNA levels (Fig. 4E). Consistent with our hypothesis that TUBB2B binds to and enhances stability of eEF1A1, treatment of cells with proteasome inhibitor MG132 prevented the reduction of eEF1A1 by TUBB2B silencing (Fig. 4F). Given that eEF1A1, as a key factor of translation elongation, is critical for protein synthesis in eukaryotic cells, we further analyzed the impact of TUBB2B depletion on protein translation using a puromycin-based incorporation assay. In both TNBC cell lines, TUBB2B depletion decreased global protein synthesis (Fig. 4G). Next, we investigated if TUBB2B regulates TNBC spheroid growth via eEF1A1. In control cells expressing empty vector CD532A-1, knockdown of TUBB2B significantly decreased 3D spheroid size (Fig. 4H-J). In contrast, overexpression of eEF1A1 restored spheroid growth in TUBB2B-depleted cells (Fig. 4H-J). Taken together, these studies identified eEF1A1 as an interacting partner of TUBB2B, and a novel role of TUBB2B in regulating protein translation.

**Fig. 5:**
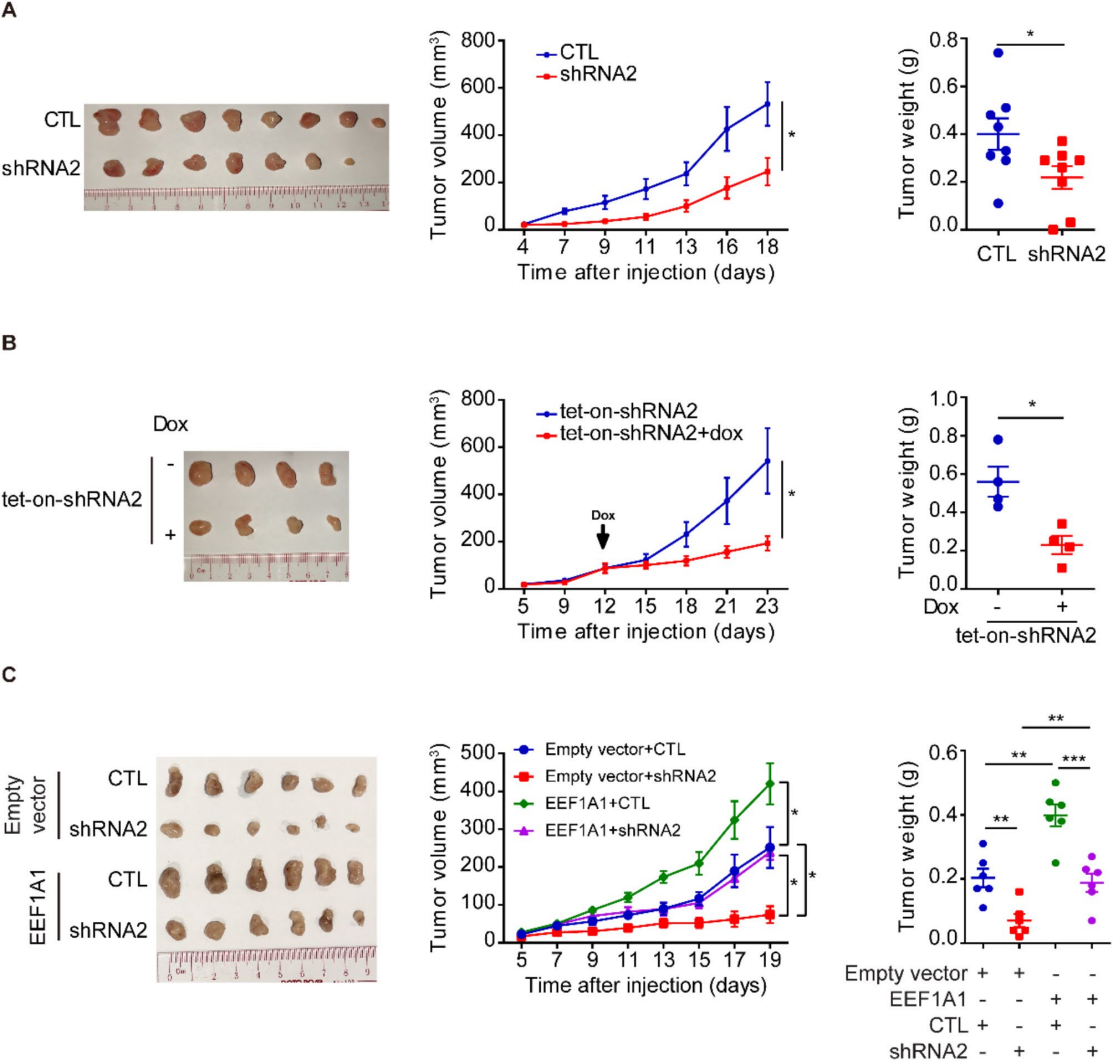
Depletion of TUBB2B suppresses TNBC tumor growth. (**A**) HCC1806 cells infected with lentiviral vector encoding control or TUBB2B shRNA2 were injected orthotopically in the MFP of nude mice. *n* = 8. Left panel: image of tumors. Graphs showing tumor growth (middle panel) and tumor weight (right panel). (**B**) HCC1806 cells with tet-on TUBB2B shRNA2 were injected orthotopically in the MFP of nude mice. *n* = 4. Treatment of dox-contained drinking water started at the indicated time. Left panel: image of tumors. Graphs showing tumor growth (middle panel) and tumor weight (right panel). (**C**) HCC1806 cells with empty vector or eEF1A1 overexpression were infected with lentiviral vector encoding control or TUBB2B shRNA2 and then injected orthotopically in the MFP of nude mice. *n* = 6. Left panel: image of tumors. Graphs showing tumor growth (middle panel) and tumor weight (right panel). All error bars represent SEM, two-tailed Student t-test (* *P* < 0.05, ** *P* < 0.01, *** *P* < 0.001)

### Depletion of TUBB2B inhibits TNBC tumor growth and brain metastasis colonization in vivo

To examine the role of TUBB2B in TNBC oncogenesis, we extended our studies to in vivo models by implanting TNBC cells orthotopically. Compared to control HCC1806 xenografts, the growth of TUBB2B-depleted tumors was significantly reduced (Fig. 5A). In the tumor maintenance model, HCC1806 cells with tet-on TUBB2B shRNA were implanted orthotopically to generate tumor-bearing mice, followed by dox treatment to induce gene knockdown. TUBB2B silencing in the tumor-bearing mice profoundly reduced tumor volume and weight (Fig. 5B). We also examined the role of eEF1A1 in mediating TUBB2B-promoting tumor growth in vivo, and showed that overexpression of eEF1A1 could partially rescue the tumor growth in TUBB2B-depleted cells (Fig. 5C). These findings point to the therapeutic potential of targeting TUBB2B in established TNBC tumors.

**Fig. 6:**
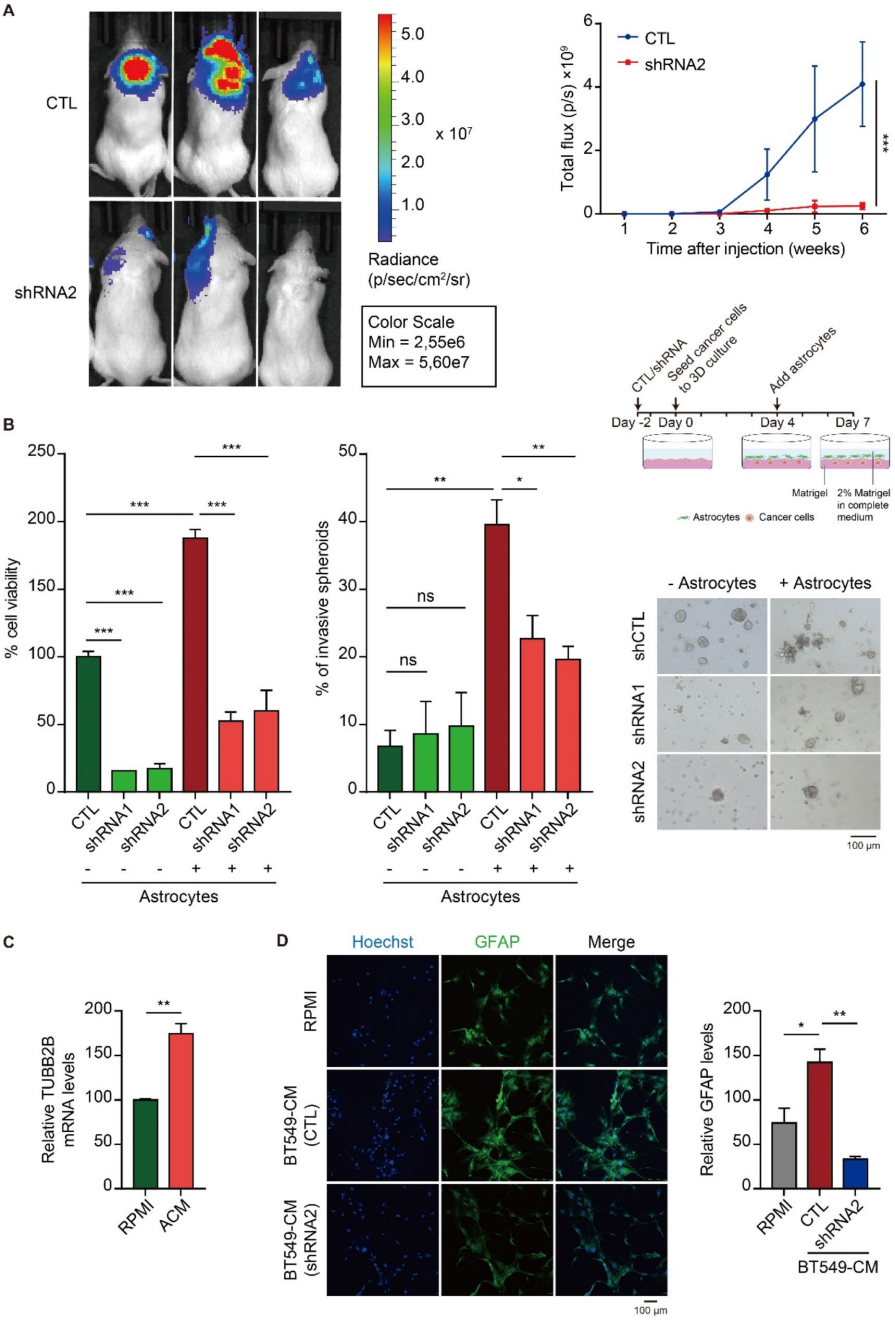
TUBB2B silencing inhibits TNBC outgrowth in the brain. (**A**) Firefly luciferase-expressing HCC1806 cells containing control or TUBB2B shRNA2 were implanted into the brains of NSG mice via ICA injection. Tumor burden was assessed by whole-body bioluminescence at the indicated time points after cell implantation. Representative images of mice at the end of experiments are shown (left). Quantification of total bioluminescence photon flux formed by HCC1806 cells at the indicated time after tumor inoculation is depicted in the graph (right). (**B**) Schematics of co-culture model of TNBC cells and astrocytes. HCC1143 containing TUBB2B shRNA or control were co-cultured with astrocytes or cultured alone. The cell viability was assessed by 3D cell titer-Glo assay. The percentage of invasive spheroids was quantified. Representative images are shown. (**C**) HCC1806 cells treated with ACM were subjected to RT-qPCR to analyze TUBB2B mRNA levels. (**D**) Astrocytes were treated with BT549-CM from BT549 cells expressing TUBB2B shRNA2 or control shRNA. IF was performed using GFAP antibody. Nuclei were labelled with Hoechst. Images were captured by confocal microscopy. Relative GFAP levels were quantified and depicted in the bar graph. All error bars represent SEM, two-tailed Student t-test (* *P* < 0.05, ** *P* < 0.01, *** *P* < 0.001)

The brain is one of the most common sites for TNBC metastasis. Based on bioinformatics analysis of public datasets as well as our in-house RNAscope data, TUBB2B is upregulated in brain metastasis (Fig. 1B and C), and its high expression predicts poorer DMFS in breast cancer patients (Fig. 1G). Therefore, we further determined the role of TUBB2B in TNBC brain metastatic growth. We have set up an in vivo model for brain metastasis using an improved intracarotid injection method [27] where brain metastases were generated in different regions of the brain, including the striatum, hippocampus, cortex and thalamus. We showed that depletion of TUBB2B in TNBC cells almost completely inhibited brain metastasis colonization (Fig. 6A). To recapitulate the brain metastatic niche, we developed an in vitro microfluidic device designed to culture cancer spheroids and astrocytes in a spatially organized manner (Fig. S3A). Astrocytes are glial cells that comprise approximately 50% of brain cells and are implicated in tumor aggressiveness in the brain microenvironment [28]. The cancer cells were cultured in the middle two channels in Matrigel, and astrocytes were cultured in the side two channels in 2D (Fig. S3A). The invasiveness and growth of the TNBC spheroids were assessed by quantifying the invasive area, spheroid number, and spheroid relative area under both TUBB2B knockdown and eEF1A1 overexpression conditions (Fig. S3B). The results showed that the depletion of TUBB2B in TNBC cells inhibited the invasiveness of spheroids and their growth in the brain metastatic niche. Importantly, the inhibition was rescued by overexpression of eEF1A1 (Fig. S3B).

Intrigued by the potent effect of TUBB2B on brain metastases, we next investigated the role of TUBB2B in the interplay between TNBC cells and the brain microenvironment. TNBC cells were seeded onto Matrigel to form spheroids for 4 days, followed by seeding astrocytes on top of Matrigel for co-culturing for 3 days, where astrocytes and TNBC cells were not in direct contact. Compared to TNBC spheroids alone, co-cultured astrocytes significantly enhanced TNBC growth and invasiveness (Fig. 6B). Importantly, these promoting effects of astrocytes on TNBC cells were abrogated by TUBB2B depletion in tumor cells (Fig. 6B).

Based on these data, we hypothesized that there is a positive feedback loop between astrocytes and TUBB2B in TNBC cells. Indeed, TUBB2B expression in both HCC1806 and BT549 cells was potentiated when treated with astrocyte-conditioned medium (ACM; Fig. 6C, S4A). On the other hand, as assessed by astrocyte activation marker GFAP, astrocytes were demonstrated to be activated by TNBC-conditioned medium (BT549-CM or HCC1806-CM), and the effect was diminished upon TUBB2B knockdown (Fig. 6D, S4B). These findings suggest that TUBB2B expression in TNBC cells promotes the activation of astrocytes, which in turn secretes molecules to upregulate TUBB2B in TNBC cells, resulting in increased brain metastasis colonization. To identify molecules secreted by the activated astrocytes, which up-regulate TUBB2B expression, we performed cytokine profiling. Among 36 cytokines being tested, we found that Serpin E1 and Interleukin-8 (IL-8) were the most abundant cytokines secreted in ACM. The amount of these two cytokines were furthered increased when astrocytes have been treated with HCC1806-CM (Fig. S4C). To test if these two cytokines modulate TUBB2B expression in TNBC cells, we treated HCC1806 cells with recombinant Seprin E1 and IL-8. The result showed that Serpin E1, but not IL-8 upregulated TUBB2B expression (Fig. S4D).

### Development of TUBB2B siRNA-AuNPs for inhibiting TNBC tumor growth

**Fig. 7:**
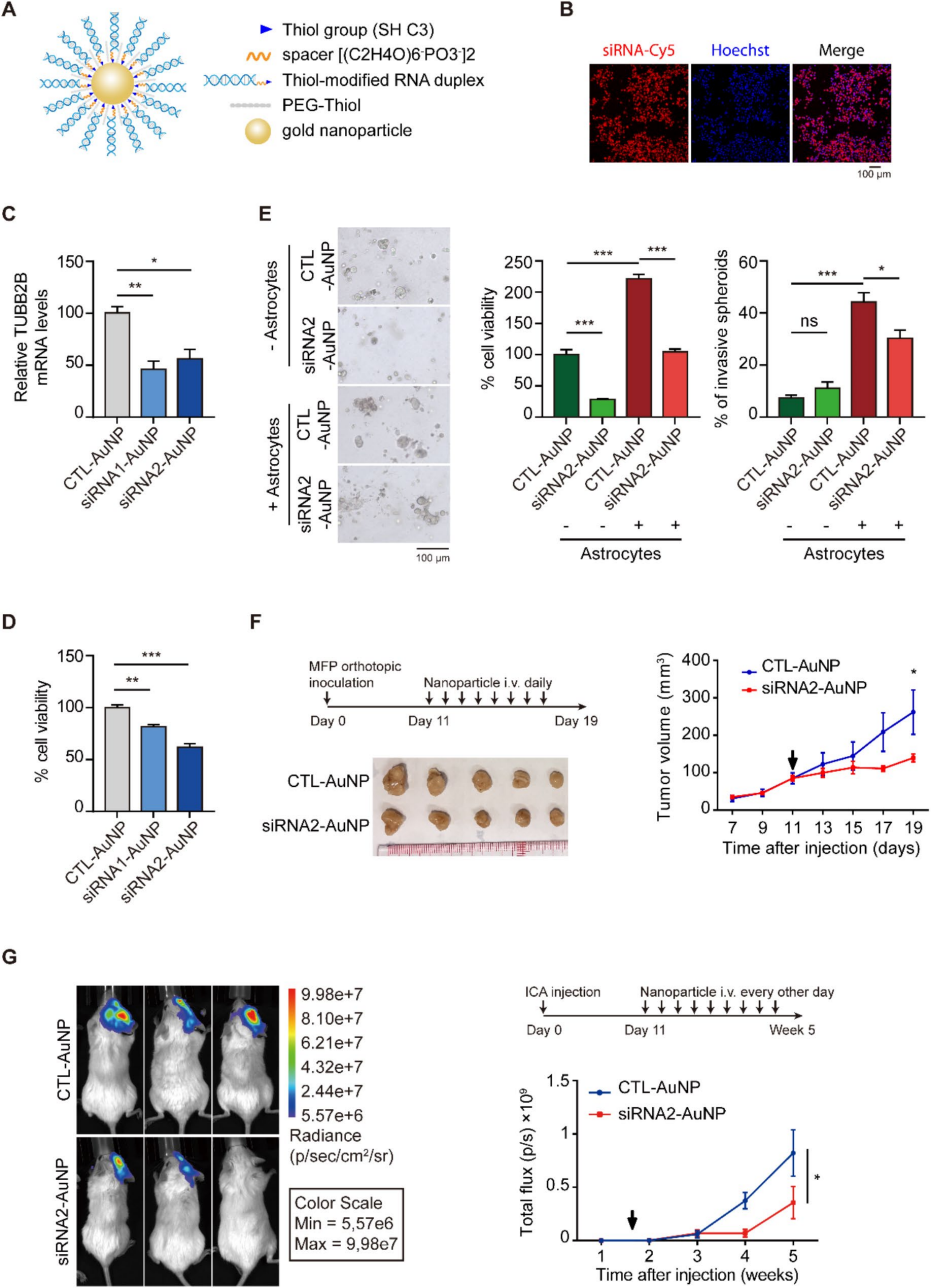
AuNPs functionalized with TUBB2B-siRNA2 inhibit TNBC cell viability and xenograft growth. (**A**) Schematic of siRNA-AuNPs. (**B**) Uptake of Cy5-labeled siRNA-AuNPs into HCC1806 cells. Cells were labeled with Hoechst to visualize nuclei. (**C**) The mRNA levels of TUBB2B were analyzed by RT-qPCR in HCC1806 cells 48 h after siRNAs-TUBB2B-AuNP or Control-AuNP treatment. (**D**) 2D cell titer-Glo assay of HCC1806 cells treated with the indicated AuNPs. (**E**) HCC1143 spheroids treated with TUBB2B-siRNA2-AuNPs or control-AuNPs were co-cultured with astrocytes or cultured alone. The cell viability was assessed by 3D cell Titer-Glo assay. The percentage of invasive spheroids was quantified. Representative images are shown. (**F**) Nude mice were orthotopically injected with HCC1806 cells in mammary fat pads to form tumors, followed by 8 intravenous injections of TUBB2B-siRNA2-AuNPs or control-AuNPs. Left panel: schematic of nanoparticle treatment and image of tumors. Graphs showing tumor growth (right panel). (**G**) Firefly luciferase-expressing HCC1806 cells were implanted into the brains of NSG mice via ICA injection, follwed by 9 intravenous injections of TUBB2B-siRNA2-AuNPs or control-AuNPs. Tumor burden was assessed by whole-body bioluminescence at the indicated time points after cell implantation. Representative images of mice at the end of experiments are shown (left). Quantification of total bioluminescence photon flux formed by HCC1806 cells at the indicated time after tumor inoculation is depicted in the graph (right). All error bars represent SEM, two-tailed Student t-test (* *P* < 0.05, ** *P* < 0.01, *** *P* < 0.001)

Recent studies have demonstrated promising applications of AuNPs in cancer therapy. This is due to their high efficiency in delivering siRNAs into tumor cells and their superior ability to cross the BBB. The safety profile of AuNPs has been supported by clinical trial data of BCL2 like 12 (Bcl2L12) siRNA-AuNP for treating glioblastoma [29], where no significant toxicity was observed in patients. Applying the same strategy, thiol-modified TUBB2B siRNAs were assembled on AuNP cores, followed by backfilling with surface-passivating polyethylene glycol (PEG) (Fig. 7A) [16]. The siRNA loading efficiency into tumor cells was confirmed using cyanine5 (Cy5)-labelled siRNA (Fig. 7B). TUBB2B siRNA-AuNPs were shown to effectively knockdown TUBB2B and reduce cell viability of TNBC cells (Fig. 7C and D). Using our TNBC spheroid-astrocyte coculture system, we also demonstrated that TUBB2B siRNA-AuNPs potently abrogated the promoting effects of astrocytes on TNBC spheroid growth and invasiveness (Fig. 7E). Importantly, our data showed that the intravenous administration of TUBB2B siRNA-AuNPs to mice resulted in a significant inhibition of tumor growth and brain metastatic colonization (Fig. 7F and G), demonstrating the preclinical efficacy of targeting TUBB2B for TNBC.

## Discussion

While luminal and HER2-overexpressed subtypes of breast cancer have seen various treatment options in recent years, TNBC has minimal effective targeted therapy. In addition, brain metastasis is commonly observed in TNBC patients, with an increased frequency in young and premenopausal patients. Despite advancements in radiotherapy and neurosurgery, more than 50% of patients die from their brain metastases. Therefore, we aimed to identify novel molecular targets for therapeutic intervention in TNBC growth and brain metastasis colonization. Our bioinformatics analysis revealed TUBB2B as a potential cancer driver gene in TNBC. We demonstrated that TUBB2B is overexpressed in approximately 26% of TNBC cases, and its upregulation predicts poor DMFS in breast cancer patients. TUBB2B is highly expressed in neuronal progenitors during embryonic development and plays a critical role in neuronal development. In postnatal mouse brains, TUBB2B is no longer expressed in cortical neurons but persists in the glial lineage [30]. Consistent with its essential roles in neuronal migration and development, loss of TUBB2B has been implicated in rare forms of congenital neuronal disorders, including polymicrogyria, microcephaly and axon guidance defects [7]. Neurological diseases due to the overexpression of TUBB2B have not been reported, but a recent study showed that TUBB2B is upregulated in astrocytes of patients with Alzheimer’s disease (AD). However, the functional role of TUBB2B in AD or other neuroinflammatory phenotypes, remains to be determined. Increasing evidence has shown the implication of TUBB2B in various malignancies. Upregulated TUBB2B in neuroblastoma and endometrial cancer is associated with poorer prognosis [9, 13]. A recent study has demonstrated that TUBB2B promotes HCC pathogenesis by modulating cytochrome P450 family 27 subfamily A member 1 (CYP27A1) expression and cholesterol metabolism [14]. Although overexpression of TUBB2B has been found in endocrine therapy-resistant breast cancer and brain metastases of TNBC patients [10, 11], its functional role in breast cancer or metastasis has not been identified.

In this study, we uncovered an oncogenic role of TUBB2B in breast cancer by observing that depletion of TUBB2B induces TNBC cell apoptosis and inhibits tumor maintenance and brain metastasis colonization in preclinical models. Our findings suggest that targeting TUBB2B therapeutically would be promising for TNBC patients, as it has minimal effect on normal breast cells or luminal breast tumor cells upon TUBB2B silencing. In addition to the translational prospect, one of our important findings is the identification of eEF1A1 as a novel interacting partner of TUBB2B. The roles of TUBB2B have primarily been studied in cortical development during the embryonic stage, and its molecular function is largely attributed to its role as a component of the microtubule cytoskeleton. The interacting partners of TUBB2B have not been systematically explored. Through IP followed by MS, we identified numerous TUBB2B-associated proteins in TNBC cells, including eEF1A1, myosin heavy chain 9 (MYH9), and heterogeneous nuclear ribonucleoprotein A2/B1 (HNRNPA2B1). Some of these candidates, including eEF1A1, are known microtubule-associated proteins [31], validating our MS approach. eEF1A1 plays essential roles in many cellular processes, including cell growth and apoptosis [21]. eEF1A1 has been shown to be highly expressed in various cancers. In HCC and B cell lymphoma, overexpression of eEF1A1 predicts poor patient survival [23, 32]. Furthermore, eEF1A1 is one of ribosome pathway genes enriched in circulating tumor cells of colorectal cancer [24]. eEF1A1 also contributes to therapeutic resistance in prostate cancer [25]. In breast cancer, eEF1A1 is one of the six signature genes that predict lymph node metastasis [33], and high expression of eEF1A1 protects tumor cells from stress-induced cell death [26]. Indeed, knockdown studies demonstrated that eEF1A1 depletion led to reduced HCC cell viability [22] and diminished breast cancer cell invasiveness [34]. Based on these observations, we prioritized focusing on identifying eEF1A1 as an interacting partner of TUBB2B, and determining their roles in TNBC cell survival. We first confirmed the interactions between TUBB2B and eEF1A1 in TNBC cells and demonstrated the enhancement of eEF1A1 stability by TUBB2B. It is noteworthy that although several mechanisms have been identified for the regulation of eEF1A1 mRNA expression, including the binding of long non-coding RNA (lncRNA) metastasis associated lung adenocarcinoma transcript 1 (MALAT1) and Myc to the eEF1A1 promoter [35, 36], proteins that regulate the stability of eEF1A1 have not been well-studied. There is one report showing that ubiquitin-like protein FAT10 could stabilize eEF1A1 and promote cancer cell proliferation [37]. To the best of our knowledge, this study is the first to show a microtubule protein that binds to and regulates the stability of eEF1A1. Based on the well-known function of eEF1A1, we further identified a novel function of TUBB2B in regulating the protein translation machinery. Ectopic overexpression of eEF1A1 rescued the altered phenotype caused by TUBB2B knockdown, indicating that TUBB2B protects TNBC cells from apoptosis via eEF1A1.

The study of brain metastasis has been hindered by the lack of robust preclinical models. Nevertheless, recent work has begun to shed light on the molecular mechanisms of brain metastasis colonization. After crossing the BBB, tumor cells interact with various stromal cells in the brain parenchyma, including microglia, neurons, endothelial cells, and astrocytes [38]. In normal physiological conditions, astrocytes play crucial roles in maintaining the central nervous system (CNS) homeostasis and BBB, supporting neuronal function, as well as injury responses [39]. Cytokines such as Interleukin-6 (IL-6), C-C motif chemokine 5 (CCL5) and tumor necrosis factor alpha (TNFα) are key mediators of astrocyte activation [40]. These cytokines activate various signaling pathways including JAK/STAT3, PI3K/Akt and NFκB signaling, which in turn enhance astrocyte growth and survival and the production of additional inflammatory mediators. Other stimuli that activate astrocytes include neurotransmitters, environmental chemicals, extracellular ions, and metabolites. Activated astrocytes support neuronal survival by releasing various neurotrophic factors and regulate synaptic transmission and plasticity by modulating extracellular ion concentration. In pathological conditions such as AD and Parkinson’s disease, chronic release of pro-inflammatory cytokines by astrocytes can exacerbate neuronal damage. In addition, dysregulated astrocytic glutamate uptake and potassium channel dysfunction in astrocytes have been shown to play a key role in neurodegeneration. Reactive astrocytes are also implicated in the formation of glial scar following brain injury [39, 41]. In the context of brain metastasis, reactive astrocytes serve as the first host defense by producing plasmin. Interestingly, tumor cells can secrete serpins including neuroserpin and serpin B2, inhibitors of plasminogen activator, to promote their survival [42]. In contrast to their anti-tumor function, emerging evidence has shown cancer-promoting roles for astrocytes. For example, astrocytes can induce PTEN loss in metastatic TNBC cells via exosomal microRNA, resulting in brain metastasis outgrowth [11]. In addition, a subpopulation of STAT3 + reactive astrocytes were reported to drive a pro-metastatic environment. In fact, promising results were observed in a clinical trial where blocking STAT3 signaling significantly inhibited brain metastasis [43]. In the present study, we established a robust brain metastatic colonization model and demonstrated the critical role of TUBB2B in driving brain metastatic growth. These findings align with the well-known function of TUBB2B in CNS and suggest that TNBC cells adopt brain-like properties by overexpressing TUBB2B. Interestingly, another brain-specific tubulin isoform, TUBB3 has been shown to promote breast cancer brain metastatic potential without affecting the growth of the primary tumor, likely by enhancing the invasive properties of breast cancer cells [44]. Using co-culture studies, our data highlight the interplay between tumor cells and the microenvironment by demonstrating the pro-metastatic function of astrocytes for TNBC cells. We showed that overexpression of TUBB2B in TNBC cells promotes the activation of astrocytes, which in turn secrete molecules such as Serpin E1 to upregulate TUBB2B in TNBC cells, forming a positive feedback loop to enhance colonization of TNBC cells in the brain.

In addition to its canonical function in protein synthesis, eEF1A1 has been shown to regulate various specific genes involved in neurogenesis and other processes in the CNS. For example, deacetylation of eEF1A1 modulates the activation of Sox10-target genes and promotes CNS remyelination [45]. In addition, eEF1A proteins play an important role in axonal repair via mTOR pathway after CNS injury [46]. eEF1A1 is also implicated in neuroinflammation of AD and Parkinson’s Disease. Interestingly, it regulates IL6 and CCL5 expression in glioma cells, exacerbating the neuroinflammatory process [47]. Both IL6 and CCL5 are known cytokines that activate astrocytes. It would be interesting to test in future studies if eEF1A1 mediates the activation of astrocytes by TUBB2B in our brain metastasis models.

siRNA has emerged as a powerful technology for silencing specific genes by degrading mRNA and is being explored widely as a therapeutic approach for various diseases. The Food and Drug Administration (FDA) has recently approved four siRNA medications, and many siRNA-based therapeutics have entered clinical investigations [48]. siRNA has also been applied as a novel cancer therapy, particularly for genes that encode “undruggable” targets. However, the vulnerability of siRNA to degradation inside the body remains a major issue in delivery. AuNPs have been shown to enhance siRNA stability and circulation half-life and effectively cross BBB to deliver siRNAs to intracranial tumors. In particular, an siRNA-based AuNP therapeutic agent (drug moniker: NU-0129) is currently being tested for treating glioblastoma in a phase 0 first-in-human trial (NCT03020017) [29]. Given the high expression of TUBB2B in primary TNBC tumors and brain metastases, in this study we used AuNPs as the delivery platform for TUBB2B siRNA to conduct proof-of-concept studies for future clinical applications. We are excited to demonstrate potent inhibition of TNBC growth as well as brain metastatic colonization when mice were treated with TUBB2B siRNA-AuNPs.

## Conclusions

In summary, we identified TUBB2B as a critical oncogenic gene that promotes primary TNBC growth and brain metastatic colonization. We also uncovered eEF1A1 as an interacting partner of TUBB2B and a novel function of TUBB2B in protein translation. The upregulation of TUBB2B in TNBC in the brain microenvironment forms a positive feedback loop with astrocytes, promoting the colonization of TNBC cells. Considering its clinical significance and minimal expression of TUBB2B in normal breast epithelial cells, targeting TUBB2B would be a promising strategy to treat patients with TUBB2B-high-expressing TNBC.

## Electronic supplementary material

Below is the link to the electronic supplementary material.

Supplementary Material 1

Supplementary Material 2

## Data Availability

The data generated in this study are available upon request from the corresponding author.

## Abbreviations

2D: Two-dimensional
3D: Three-dimensional
ACM: Astrocyte-conditioned medium
AD: Alzheimer’s disease
ATCC: American Type Culture Collection
AuNP: Gold nanoparticle
BBB: Blood-brain barrier
Bcl2L12: BCL2 like 12
BT549-CM: BT549-conditioned medium
CCL5: C-C motif chemokine 5
CDS: Coding DNA sequence
CNS: Central nervous system
co-IP: Co-immunoprecipitation
Cy5: Cyanine5
CYP27A1: Cytochrome P450 family 27 subfamily A member 1
DMEM: Dulbecco’s Modified Eagle medium
DMFS: Distant metastasis-free survival
Dox: Doxycycline
eEF1A1: Eukaryotic translation elongation factor 1 alpha 1
FBS: Fetal bovine serum
FDA: Food and Drug Administration
FFPE: Formalin-fixed paraffin-embedded
GAPDH: Glyceraldehyde-3-phosphate dehydrogenase
GEO: Gene Expression Omnibus
GFAP: Glial fibrillary acidic protein
HCC: Hepatocellular carcinoma
HCC1806-CM: HCC1806-conditioned medium
HER2: Human epidermal growth factor receptor 2
HMEC: Human mammary epithelial cell
HNRNPA2B1: Heterogeneous nuclear ribonucleoprotein A2/B1
HRP: Horseradish peroxidase
ICA: Internal carotid arteries
IF: Immunofluorescence
IL-6: Interleukin-6
IL-8: Interleukin-8
IP: Immunoprecipitation
lncRNA: Long non-coding RNA
MALAT1: Metastasis associated lung adenocarcinoma transcript 1
MFP: Mammary fat pad
MLKL: Mixed lineage kinase domain like pseudokinase
MS: Mass spectrometry
MYH9: Myosin heavy chain 9
NAFLD-HCC: Non-alcoholic fatty liver disease-associated hepatocellular carcinoma
NSG: NOD scid gamma
OS: Overall survival
PARP: Poly(ADP-ribose) polymerase 1
PBS: Phosphate-buffered saline
PDMS: Poly(dimethylsiloxane)
PEG: Polyethylene glycol
PEG-SH: PEG-Thiol
PI: Propidium Iodide
RISH: RNA in situ hybridization
RPMI: Roswell Park Memorial Institute 1640 medium
RT-qPCR: Reverse transcription-quantitative polymerase chain reaction
SDS-PAGE: Sodium dodecyl sulfate-polyacrylamide gel electrophoresis
SEM: Standard error of the mean
shRNAs: Short hairpin RNAs
siRNA-AuNP: siRNA-gold nanoparticle
TCGA: The Cancer Genome Altlas
TNBC: Triple-negative breast cancer
TNFα: Tumor necrosis factor alpha
TUBB: Tubulin beta class I
TUBB2A: Tubulin beta 2A class IIa
TUBB2B: Tubulin beta 2B class IIb
TUBB2C: Tubulin beta 2C class IIc
TUBB3: Tubulin beta 3 class III
TUBB4B: Tubulin beta 4B class IVb
WB: Western Blot
