# Supplementary material for: Targeting TUBB2B inhibits triple-negative breast cancer growth and brain-metastatic colonization
Source: J Exp Clin Cancer Res. 2025 Feb 17;44:55. doi: 10.1186/s13046-025-03312-y (PMC11831766; doi:10.1186/s13046-025-03312-y)
Supplement: Supplementary file 2 — Supplementary Material 2 [file 13046_2025_3312_MOESM2_ESM.pdf]

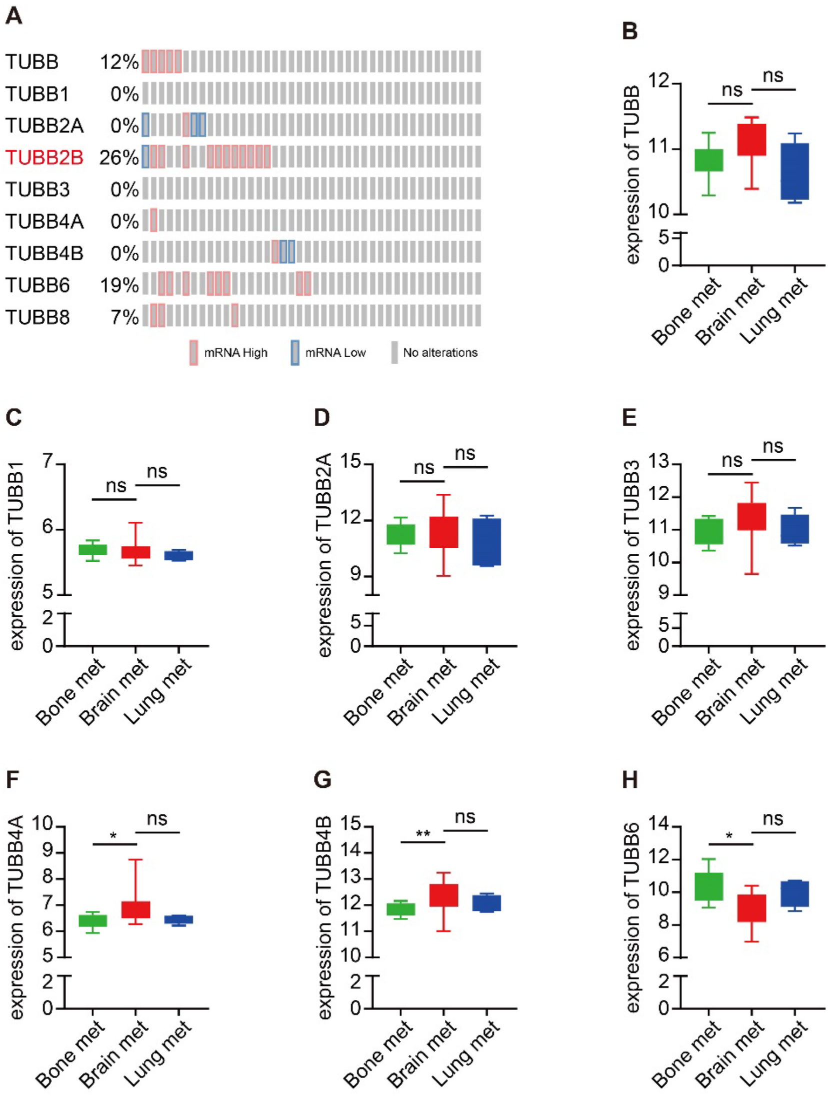

Figure S1. TUBB2B is highly expressed in TNBC primary tumors and brain metastases. **(A)** OncoPrint graph summarizing mRNA alteration of the β-tubulin isoforms in 82 TNBC patients from TCGA dataset [15] (accessed through the cBioPortal database), including mRNA high (red), mRNA low (blue), and no alteration (grey). **(B-H)** Graphs showing the expression value of β-tubulin isoforms, including TUBB **(B)**, TUBB1 **(C)**, TUBB2A **(D)**, TUBB3 **(E)**, TUBB4A **(F)**, TUBB4B **(G)**, TUBB6 **(H),** in brain, bone and lung metastases of breast cancer. Data are from Human Cancer Metastasis Database; GSE14017(n=29). All error bars represent SEM, two-tailed Student t-test (* P<0.05, ** P<0.01).

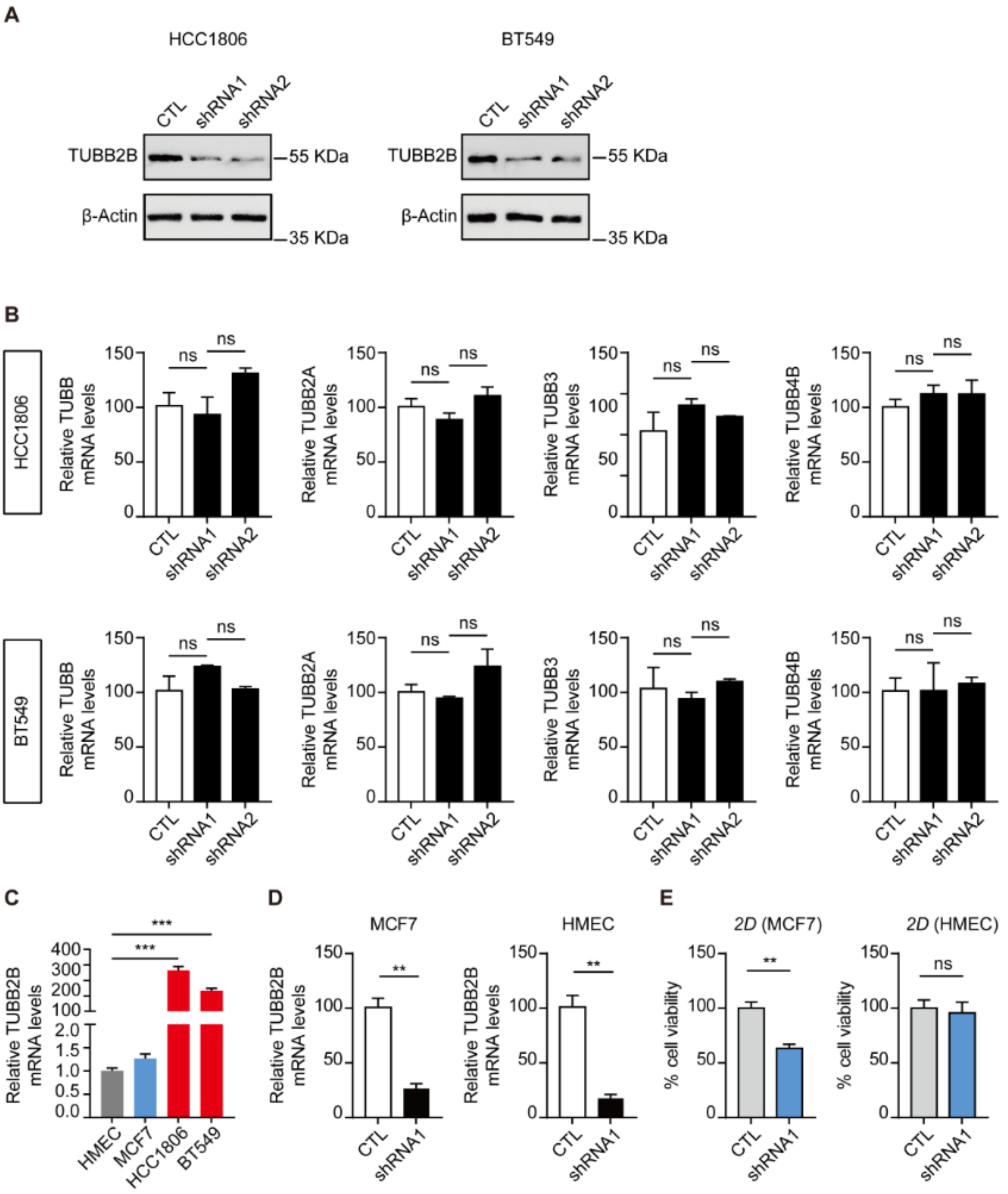

Figure S2. The efficacy of TUBB2B knockdown by shRNA. **(A)** The protein levels of TUBB2B in HCC1806 and BT549 cells infected with control or TUBB2B shRNA1/2 lentiviral vector were analyzed by WB. **(B)** mRNA levels of different β-tubulin isoforms (TUBB, TUBB2A, TUBB3, TUBB4B) were analyzed by RT-qPCR in HCC1806 and BT549 cells infected with control or TUBB2B shRNA1/2 lentiviral vector. **(C)** mRNA levels of TUBB2B were analyzed by RT-qPCR in the indicated cell lines. **(D)** mRNA levels of TUBB2B in MCF7 cells and HMEC with control or TUBB2B shRNA1 were analyzed by RT- qPCR. **(E)** 2D cell Titer-Glo assay of MCF7 cells and HMEC. All error bars represent SEM, two-tailed Student t-test (** P<0.01, (*** P<0.001).

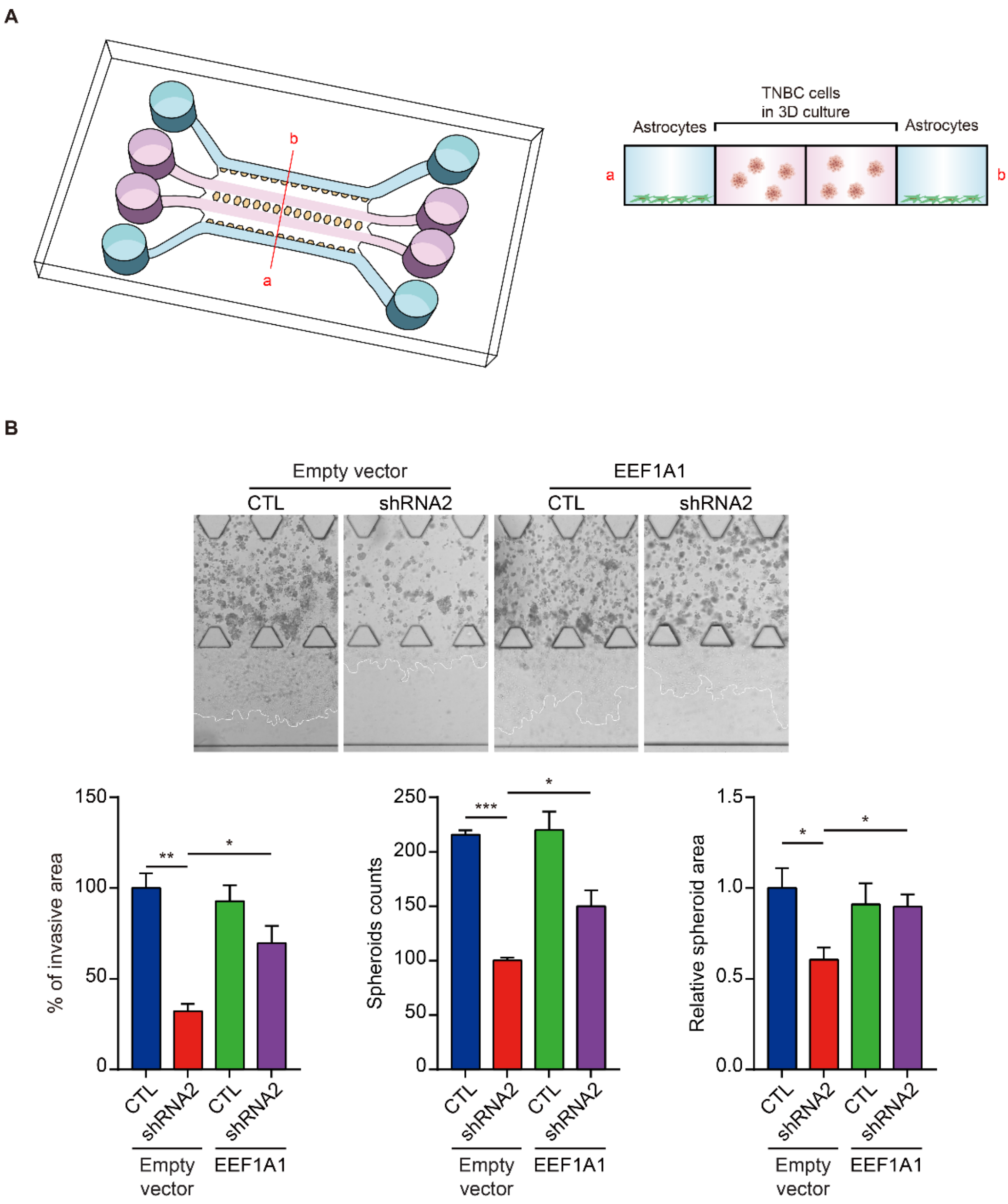

Figure S3. A microfluidic device for recapitulating the brain metastatic niche. **(A)** Schematic illustration of the microfluidic device with 4 channels and its cross-sectional illustration with different cell types in channels. **(B)** Images of HCC1806 tumor spheroids with TUBB2B knockdown and/or eEF1A1 overexpression co-cultured with astrocytes in microfluidic devices (upper panel). The invasive area, spheroid number, and relative spheroid area were quantified (bottom panel). All error bars represent SEM, two-tailed Student t-test (* P<0.05, ** P<0.01, *** P<0.001).

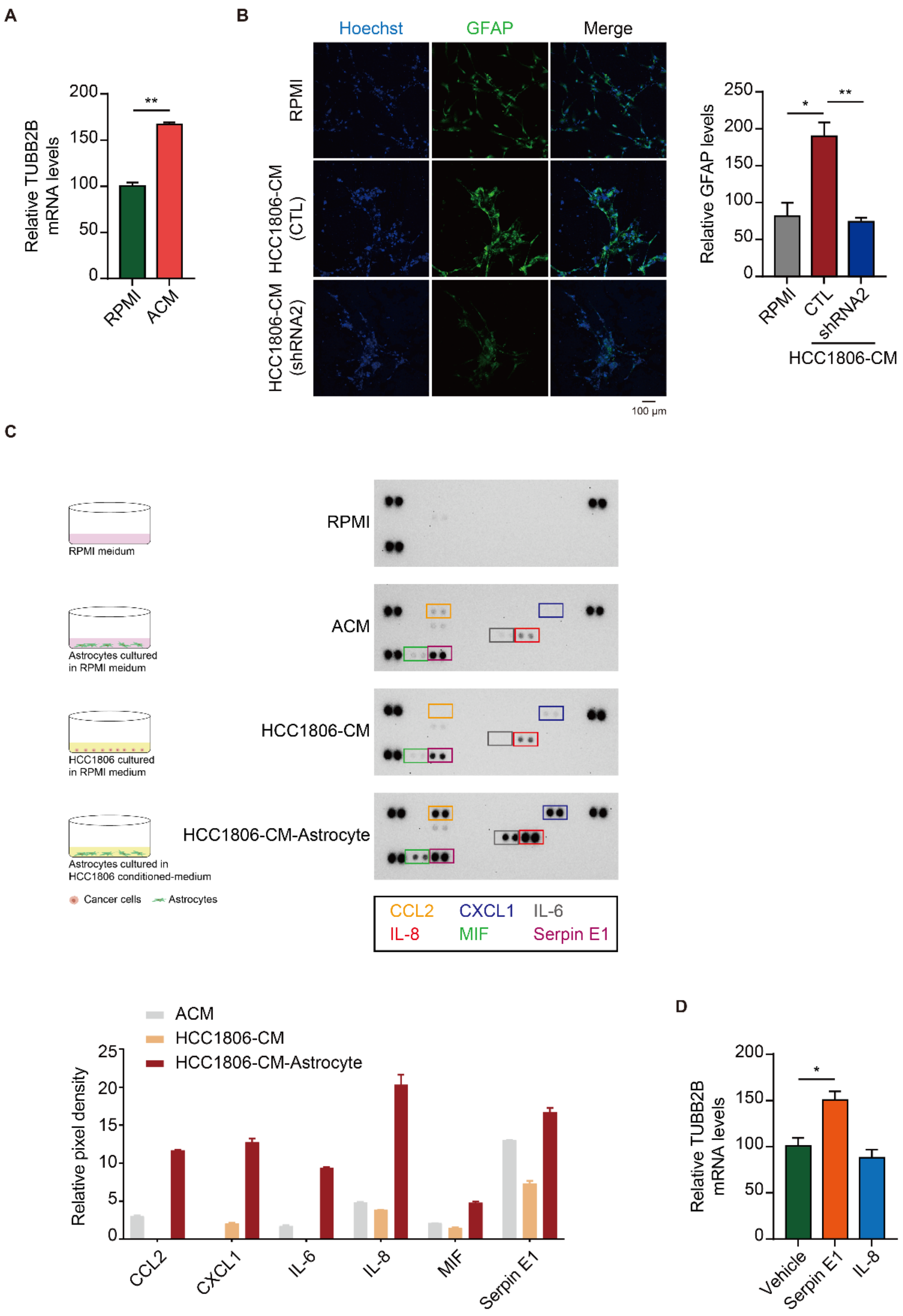

Figure S4. Secretome profiling of human astrocytes. **(A)** BT549 cells treated with ACM were subjected to RT-qPCR to analyze TUBB2B mRNA levels. **(B)** Astrocytes were treated with

HCC1806-CM from HCC1806 cells expressing TUBB2B shRNA2 or control shRNA. IF was performed using GFAP antibody. Nuclei were labelled with Hoechst. Images were captured by confocal microscopy. Relative GFAP levels were quantified and depicted in the bar graph. **(C)** Secretome profiling of human astrocytes. Upper left: schematic showing the conditioned medium collection. From top to bottom: RPMI control, Astrocytes cultured in RPMI medium, HCC1806 cultured in RPMI medium, Astrocytes cultured in HCC1806 conditioned RPMI medium. Upper right: blots showing the expression of cytokines and chemokines in each conditioned medium. Bottom: the relative pixel density of each dot was quantified by ImageJ software. **(D)** HCC1806 cells treated with Serpin E1 (5 ng/ml) or IL-8 (5 ng/ml) were subjected to RT-qPCR to analyze TUBB2B mRNA levels. All error bars represent SEM, two-tailed Student t-test (* $P<0.05$, ** $P<0.01$, *** $P<0.001$).
